# Translational co-regulation of a ligand and inhibitor by a conserved RNA element

**DOI:** 10.1093/nar/gkx938

**Published:** 2017-10-20

**Authors:** Andreas Zaucker, Agnieszka Nagorska, Pooja Kumari, Nikolai Hecker, Yin Wang, Sizhou Huang, Ledean Cooper, Lavanya Sivashanmugam, Shruthi VijayKumar, Jan Brosens, Jan Gorodkin, Karuna Sampath

**Affiliations:** Cell & Developmental Biology Unit, Division of Biomedical Sciences, Warwick Medical School, University of Warwick, Coventry, CV4 7AL, UK; Center for non-coding RNAs in Technology and Health, Department of Veterinary and Animal Sciences, Faculty for Health and Medical Sciences, University of Copenhagen, Grønnegårdsvej 3, 1870 Frederiksberg C, Denmark

## Abstract

In many organisms, transcriptional and post-transcriptional regulation of components of pathways or processes has been reported. However, to date, there are few reports of translational co-regulation of multiple components of a developmental signaling pathway. Here, we show that an RNA element which we previously identified as a dorsal localization element (DLE) in the 3′UTR of zebrafish *nodal-related1/squint* (*ndr1/sqt*) ligand mRNA, is shared by the related ligand *nodal-related2/cyclops (ndr2/cyc)* and the nodal inhibitors, *lefty1* (*lft1*) and *lefty2* mRNAs. We investigated the activity of the DLEs through functional assays in live zebrafish embryos. The *lft1* DLE localizes fluorescently labeled RNA similarly to the *ndr1/sqt* DLE. Similar to the *ndr1/sqt* 3′UTR, the *lft1* and *lft2* 3′UTRs are bound by the RNA-binding protein (RBP) and translational repressor, Y-box binding protein 1 (Ybx1), whereas deletions in the DLE abolish binding to Ybx1. Analysis of zebrafish *ybx1* mutants shows that Ybx1 represses *lefty1* translation in embryos. CRISPR/Cas9-mediated inactivation of human YBX1 also results in human NODAL translational de-repression, suggesting broader conservation of the DLE RNA element/Ybx1 RBP module in regulation of Nodal signaling. Our findings demonstrate translational co-regulation of components of a signaling pathway by an RNA element conserved in both sequence and structure and an RBP, revealing a ‘translational regulon’.

## INTRODUCTION

The fate of mRNAs in the cytoplasm is to a large extent controlled by RBPs effecting translation, localization and stability of the mRNA targets (1). These processes regulate gene expression, ultimately by controlling the amount of protein that is produced. The regulation of gene expression at the level of RNAs is based on the binding of *cis*-regulatory recognition elements by *trans*-acting factors including RBPs and other RNAs.

Whereas transcription factors recognize binding sites in the genome that are typically short DNA sequence motifs, RBPs are thought to utilize sequence as well as structural information to identify target RNAs. Accordingly, the nature of RNA elements is quite diverse, ranging from sequences of a certain nucleotide composition (e.g. pyrimidine-rich sequences), and specific sequence motifs, to structural motifs, or combinations of sequence and structure motifs. AU-rich elements (AREs) are destabilizing sequences in short-lived RNAs. They are composed of repeats of an AUUUA pentamer in an AU context. More than 4000 human transcripts contain a putative ARE (2). The recognition of the ARE by binding proteins typically has destabilizing consequences for the bound RNA, but can also have a stabilizing effect: for instance, upon binding by HuR/ELAV1 (3). In baker's yeast, the RNA sequence ‘UGUANAUA’ is bound by Puf3p, a member of the Pumilio/fem-3 mRNA binding factor (PUF) family of sequence specific RBPs (4). Many RNA elements that control the localization of transcripts in *Drosophila* form stem-loop structures: the *bicoid* localization element 1 (BLE1) (5), the transport/localization sequence (TLS) in *K10* and *orb* mRNAs (6), the apical localization element (WLE3) in *wingless* mRNA (7), and the *hairy* localization element (HLE) (8). The *K10* TLS is primarily a structural motif, and sequence seems to play a negligible role for its function (9,10). However, for other localization elements to be fully functional, e.g. the WLE3, both specific structural and sequence features are required (7,11). Elements in RNAs are typically recognized and regulated by RBPs (12). More than 1500 RBPs have been identified in the human genome (13). However, only a handful of RNA-binding domains are known. Moreover, the binding sites for most RBPs are not known and the basis of their interactions with their target RNAs is poorly understood (13).

We previously showed that the 3′UTR of zebrafish *nodal-related1/squint* mRNA (*ndr1/sqt*, hereafter *sqt*) contains an RNA element that directs maternal *sqt* RNA localization into one or two cells in four-cell stage embryos (14). Maternal *sqt* RNA asymmetrically localizes to the presumptive dorsal cells of the embryo (14). Accordingly, the RNA element was named ‘dorsal localization element’, DLE. The DLE was mapped by phylogenetic foot-printing of the nodal 3′UTR from a variety of cyprinid species, closely or distantly related to zebrafish. Through mutational analyses and functional assays in zebrafish embryos, the DLE was found to be a bipartite element composed of a short sequence motif followed by a structural feature (short hairpin/stem-loop) (15). The trans-acting factor that binds to this element is a conserved cold shock domain-containing protein, Y box binding protein 1 (Ybx1). Ybx1 has been implicated in many aspects of gene expression (16). Ybx1 function is required for the correct localization of *sqt* RNA in zebrafish embryos. Furthermore, the binding of Ybx1 to the *sqt* DLE leads to translational repression of *sqt* by preventing the formation of a translation pre-initiation complex. Zebrafish mutant embryos lacking Ybx1 function manifest premature *sqt* translation and Nodal signaling, mis-differentiation of embryonic progenitors and lethality. Thus, the DLE/Ybx1 is an essential localization and a translational repression module in *sqt* RNA (17).

Here, we show that in addition to *sqt/ndr1*, multiple Nodal pathway components contain a functional DLE. We demonstrate the ease of zebrafish embryo-based *in vivo* assays for functional analysis and validation of RNA elements, allowing the study of such elements in their physiological context, in the presence of cellular components that might be necessary for their activity. Our work provides evidence for co-regulation of a signaling ligand and its inhibitors by an RNA motif / RBP translational repression module, which is conserved in humans. This could be a robust mechanism for coordinating gene expression during developmental processes.

## MATERIALS AND METHODS

### Generation of constructs

Mutations in the DLE were generated by site-directed mutagenesis in the context of a full-length *lefty1* and *lefty2* cDNA cloned into pCS2+ vector, also containing a SP6 promoter and a SV40 pA, for the generation of RNA by *in vitro* transcription. The list of primers is included in Supplementary Table 1. Cyclops full-length coding sequence was amplified from zebrafish shield stage cDNA and inserted into pCS2 vector using standard methods (18). The deletion of the CA region in the *nodal-related2/cyclops* (*ndr2/cyc*) 3′UTR was made using PCR. All deletions were confirmed by sequencing.

### Zebrafish Strains

Wild type and *ybx1^sa42^* fish were maintained at 28.5°C, and embryos obtained by natural mating using standard procedures in accordance with institutional animal care regulations at the University of Warwick. To block the function of maternal Ybx1, embryos from females homozygous for a temperature sensitive mutant allele (*ybx1^sa42^*) were incubated at 28.5°C until the second cell division, shifted to 23°C until the 1000-cell stage, and returned to 28.5°C until sphere or 30% epiboly stages (17). Embryos from mating homozygous *ybx1* males with wild-type females are indistinguishable from wild-type embryos, and were used as controls.

### Fluorescent RNA synthesis and injections

Plasmid constructs were linearized using Not1 enzyme (NEB) and purified. Subsequently, one μg aliquots of template were used in transcription reactions containing 2.5 μL of Alexa 488 fluorophore UTP (0.150 mM), SP6 polymerase (Promega), m^7^G cap and NTP mix (0.5 mM rGTP, rCTP, rATP and 0.375 mM rUTP) (Promega). Un-incorporated nucleotides were removed using a micro-bio-spin column (BioRad), and fluorescent RNAs were purified using phenol-chloroform and eluted in DEPC water. Quality of the RNAs was examined on a 1.5% agarose gel, and the labeling efficiency and concentration of the RNAs was determined using a Nanodrop UV spectrophotometer (Thermo Scientific).

Aliquots of 20 pg total of each capped fluorescent mRNA were injected into embryos at the one-cell stage, and embryos were scored visually for localization at the 4-cell stage using a Nikon SMZ18 stereo-microscope. Embryos that were unfertilized or showed cleavage furrow abnormalities were discarded. To mitigate bias in analysis, two researchers independently scored the injected embryos.

For RNA co-localization assays, 10 pg green fluorescent *sqt* RNA (Alexa 488 UTP-labeled) was co-injected with 10 pg red fluorescent *lefty1* or *lefty2* RNA (Alexa 546 UTP-labeled) into 1-cell stage wild type embryos of the TU (Tuebingen) strain. Embryos were mounted in 0.7% low melting agarose, and imaged at the 4-cell stage. Z-stacks in two channels (GFP, mCherry) with a step-size of 5 μm were acquired through the whole embryo at 10× magnification on a Nikon, ECLIPSE Ni microscope equipped with a HAMAMATSU digital camera C11440, ORCA-Flash4.OLT. Maximum intensity projections were generated from the Z-stacks for both channels using Fiji software (19). Overlays of the two channels were generated to assess the extent of co-localization of the signals.

### RNA secondary structure predictions

The structure of the 88 nucleotide *sqt* DLE region was adopted from Gilligan *et al.* (15). DLE motifs were identified in the 3′UTRs of *cyc, lefty1* and *lefty2* using RNAmotif (20). Here, we searched for a pattern comprising a GCAC sequence followed by two to five intervening nucleotides, a 3 bp stem, and a variable loop. The RNAfold web-suite (21) was used for structure predictions of the 326 nt *ndr2/cyc* DLE region. The sequence used for structure prediction comprised the majority of the *ndr2/cyc* 3′UTR, and the last 50 nucleotides of the coding sequence. For analysis of formation of the DLE stem loop, we compared the probabilities for folding into the stem loop using RNAcop (22).

### Identification of conserved DLE motifs in *ndr1/sqt* orthologues

We downloaded the list of zebrafish *ndr1/sqt* (ENSDARG00000057096) orthologues from ENSEMBL (23). From this list, we selected *ndr1/sqt* orthologues in primates and rodents. Only orthologues for which ENSEMBL provides 3′UTR sequences of species that are part of the phylogenetic tree from UCSC 100-way genome alignment were considered (24). The UCSC 100-way phylogenetic tree was used to infer phylogenetic relationships in this analysis. If alternative 3′UTR sequences were available, we selected the longest 3′UTR sequence. We obtained the following 3′UTR sequences: zebrafish (ENSDART00000079692), mouse (ENSMUST00000049339), rat (ENSRNOT00000000672), marmoset (ENSCJAT00000031689), gorilla (ENSGGOT00000002595), chimp (ENSPTRT00000004869) and human (ENST00000287139). We also included 100 nucleotides of the coding sequence. We screened the sequences for DLEs using RNAMotif (20). Considering the search in orthologues sequences, we used a strict definition for a DLE. We defined a DLE motif as a GCAC sequence followed by two or three pyrimidines (U or C) and then followed by a stem loop of 3–20 bp with a 5nt to 12nt loop region for this part of the study. Next, we aligned the identified DLE sequences with the phylogeny aware multiple sequence alignment tool PRANK (25). Gaps were manually shifted for the mouse and rat sequences to match the position of the stem loop identified by RNAMotif. PETfold was then used to predict RNA secondary structures (26,27). PETfold combines evolutionary information with a thermodynamic model to predict a consensus structure.

### RNA electrophoretic mobility shift assay (EMSA)

Radioactively labeled probes for EMSAs were transcribed from PCR products with T3 RNA polymerase. Templates for probe synthesis were generated by PCR with an extended phage T3 RNA polymerase promoter (AATTAACCCTCACTAAAGGGAGAA) appended to the 5′ end of the forwards primer, and gel-purified (primers listed in Supplementary Table S1). Labeled probes were transcribed in 3 μl reactions containing 0.5 μl template, 1.5 μl αP^32^ or P^33^ UTP (3 μM), 0.6 μl 5× transcription buffer (Promega), 0.4 μl T3 polymerase (Promega), 0.2 μl RNasin (Promega), 2.5 mM rATP, rGTP and rCTP and 0.025 mM rUTP (Roche) at 37°C for 3 h. The reaction was stopped by adding 100 μl TE containing 30% glycerol and ∼0.01% Bromophenol Blue.

Full-length zebrafish Ybx1 protein was produced as a His-tagged fusion protein in BL21 cells by IPTG induction (with 0.25 mM IPTG for 12–16 h at 28°C). Recombinant Ybx1 protein was affinity purified by the His tag using Ni-NTA agarose beads (Qiagen) according to the manufacturer's instruction. The buffer was exchanged to 20 mM HEPES (pH 7.5), 200 mM NaCl by using ultra centrifugal filters (Amicon) and protein was stored in aliquots at –80°C. For gel-shifts, 1 μl of purified protein was pre-incubated with 4 μl of 2× gel-shift buffer (20 mM Hepes pH 8, 100 mM KCl, 200 mM NaCl, 0.2 mM EDTA, 20 mM DTT, 2 mM MgCl_2_, 2 mM CaCl_2_, 0.2 mM ZnSO_4_, 60% glycerol, 500 μg/ml heparin, 50 μg/ml *E.scherichia coli* tRNA). The reaction was made up to 7 μl with sterile water, incubated for 10 min at room temperature, following which 1 μl of RNA probe (∼2 nM, ∼10^5^ cpm) was added. The reaction was incubated for 20 min and loaded onto the gel, electrophoresed at 25 mA, dried, and auto-radiographed. The stacking gel was 25 mM Tris pH 6.8, 3% 19:1 acrylamide:bisacrylamide and the resolving gel was 0.5× TBE (45 mM Tris–borate pH 8.3, 1 mM EDTA), 6% 19:1 acrylamide:bisacrylamide. The anode buffer was 0.5× TBE and the cathode buffer was either 50 mM glycine, 6 mM Tris, 0.2 mM EDTA, pH 8 (for Figure 4A) or 50 mM Tricine, 5 mM Tris pH 7 (for Figure 4B and C). The tricine cathode buffer yielded better separation of protein–RNA complexes from unbound RNA and resulted in higher shifts.

For preparing embryo lysates to detect binding activity *in vivo*, embryos were homogenized in 1/10 volume lysis buffer (20 mM Tris pH 8.0, 100 mM NaCl, 0.1 mM EDTA, 1 mM 6-aminohexanoic acid, 1 mM PMSF, 25% glycerol) to make extracts. Debris was pelleted by centrifugation (20 000 × *g*, 4°C, 1 min), and supernatants were flash frozen in 50 μl aliquots in liquid N_2_. One hundred-nucleotide long probes spanning the 3′UTR of *sqt* or *lft1* were synthesized and used in RNA gel-shift assays. Templates for the probes were generated by PCR with an extended phage T3 RNA polymerase promoter (AATTAACCCTCACTAAAGGGAGAA) appended to the 5′end of the forward primer, and gel-purified. Primers are listed in Supplementary Table S1. Radioactively labeled probes were transcribed with T3 RNA polymerase (Promega, Madison, WI), mixed with extracts, and used in electrophoretic mobility-shift assays. For the competition gel-shift assays, ∼0.1 ng of radioactive probe was competed with 5, 20 or 80 ng of unlabeled RNA. Human NODAL 3′UTR probes were mixed with recombinant zebrafish Ybx1 protein or with extracts from wild-type or *ybx1* mutant embryos, and used in gel shift assays.

### RNA immunoprecipitation assay

RNA immunoprecipitation was carried out using embryo lysates as described (17). Embryos were collected at the 1 cell and 1000 cell stages, cross-linked using 1% formaldehyde and lysed in RIPA buffer (50 mM Tris–Cl pH 7.5, 1% NP-40, 0.5% sodium deoxycholate, 0.05% SDS, 1 mM EDTA, 150 mM NaCl, protease inhibitor cocktail). Anti-Ybx1 antibody (Sigma 4F12) was bound to 50 μl of magnetic beads (BioRad), incubated with 250 μl wild type embryo lysate at 4°C, washed with high stringency RIPA buffer (50 mM Tris–Cl pH 7.5, 1% NP-40, 1% sodium deoxycholate, 0.1% SDS, 1mM EDTA, 1 M NaCl, 1 M urea, protease inhibitor) and eluted with 100 μl of elution buffer (50 mM Tris–Cl pH 7, 5 mM EDTA, 10 mM DTT, 1% SDS) by heating at 70°C for 10 min. 40 μl of the eluate was used for western blot analysis and 60μl was used for RNA extraction using TRIzol reagent (Invitrogen). First-strand cDNA was synthesized using SuperScript(TM) III Reverse Transcriptase (Invitrogen), followed by RT-PCRs to detect *sqt, lefty1, lefty2* and *gapdh* transcripts. The cycle numbers for the PCR reactions were: *gapdh* 25 cycles, *sqt* 28 cycles, *lft1* 29 cycles and *lft2* 28 cycles. The list of primers is included in the Supplementary material.

### Analysis of *lefty* translation in *ybx1* mutants

Capped synthetic *lefty1-gfp* and *lefty2-gfp* reporter mRNAs were synthesized from linearized plasmid using the mMessage mMachine SP6 kit (Invitrogen, Carlsbad, CA, USA). The GFP CDS was inserted into the full-length lefty cDNA sequence, in frame right after the 3′end of the respective lefty CDS (see Figure 5A). Aliquots of 25 pg total of *lefty1-gfp* or *lefty2-gfp* RNA was injected into maternal *ybx1^sa42^* mutant embryos or control embryos (paternal *ybx1*) at the 1-cell stage as described (17). Capped lacZ RNA was injected as a control. The embryos were incubated at 28.5°C until the 4-cell stage to allow *sqt* RNA localization, shifted to 23°C until the 256-cell stage, and subsequently returned to 28.5°C until observations and scoring of GFP expression at 1000 cell (∼ 3 hpf at 28.5°C), sphere (4 hpf at 28.5°C), 30% epiboly (5 hpf at 28.5°C), and shield (6 hpf at 28.5°C) stages.

For quantitative analysis of Lefty expression, injections and temperature shifts to 23°C were done as described above, and the embryos were shifted back to 28.5°C at the 1000-cell stage (see schematic in Figure 5A). The injection solution also contained 0.5% rhodamine dextran (70 kDa), which served as an internal standard for GFP intensity measurements. Embryos were imaged at three developmental stages (1000 cell, sphere and 30% epiboly) using a Nikon, ECLIPSE Ni microscope equipped with a HAMAMATSU digital camera C11440, ORCA-Flash4.OLT. Embryos were imaged in groups of nine as a 3 × 3 array, oriented laterally in custom-made agarose-coated plates. Z-stacks of 15 optical sections were acquired (each with a 29.6 μm step size) in two channels (GFP and mCherry) at 4× magnification and 16-bit depth. Image analysis was performed using the Fiji software (19). Maximum intensity projections were generated from four consecutive optical slices with the embryos in sharp focus. Rhodamine signal in the blastoderm (embryo proper) was used to obtain sets of ROIs via threshold segmentation. The ROIs were used to measure the rhodamine and GFP signals in each embryo using the measure tool. Mean pixel intensity values were background subtracted to the values measured in un-injected control embryos. The background subtracted GFP-signals were normalized to corresponding background subtracted rhodamine signals for each embryo (GFP/RFP signal). Statistical analysis was carried out in GraphPad Prism 5.0 (GraphPad Software, Inc).

To obtain embryos from homozygous mutant mothers (M*ybx1, ybx1* mutants) or homozygous mutant fathers (P*ybx1*, controls) for the translation experiments, mutant fish were outcrossed to wild type fish of the AB strain. Homozygous *ybx1* mutant fish were maintained in the TU (Tuebingen) background.

### Phenotype Analysis of *lefty1-ΔDLE-gfp* RNA injected embryos

Aliquots of 25 pg of either *lft1-gfp* RNA or *lft1-ΔDLE-gfp* RNA were co-injected with rhodamine dextran (injection control) into 1 cell stage AB wild-type embryos. GFP and rhodamine signals were analysed for a subset of injected embryos. The embryos were incubated at 28°C until 2.5 days post fertilization and imaged using a Nikon SMZ18 microscope equipped with a Nikon DS-Fi2 color camera controlled by NIS-Elements F 4.0 software. Imaged embryos were scored by the following categories of Nodal loss-of-function phenotypes: wild type, mild, moderate, or severe. Phenotypes were independently scored by two researchers.

### CRISPR/Cas targeting of Human Ybx1

HEK293FT cells were cultured in DMEM medium with 10% FBS, and at a low passage number were transfected with a *YBX1* targeting CRISPR/Cas9 expression vector (28). Human YBX1 gRNAs were designed using the CCtop2 and CRISPRSCAN online target site prediction tools [http://crispr.cos.uni-heidelberg.de/ (29), http://www.crisprscan.org/ (30)]. The gRNAs to target human YBX1 were produced using annealed oligonucleotides, and ligated into the px459 plasmid containing the spCas9 gene and a puromycin resistance cassette. The pSpCas9(BB)-2A-Puro (PX459) V2.0 plasmid was obtained from Addgene (Addgene plasmid # 62988). Plasmids were sequenced to confirm insertion of the gRNA sequence, and subsequently transfected into HEK cells and *YBX1^−/−^* cells were selected using DMEM containing puromycin. Human YBX1 sequences were amplified from genomic DNA extracted from puromycin-selected cells by PCR and sequenced to confirm mutations in gRNA target sites.

### Western Blot Analysis

HEK293FT cells were transfected with 2 μg of plasmid at a low passage number in Opti MEM medium using Fugene reagent (Promega). After 48 h, the transfection medium was changed to DMEM with 1 μg/ml of puromycin (Gibco). After a further 24 h, the puromycin treated cells were allowed to recover in DMEM media and extracted DNA from the cells was sequenced to confirm mutations in the *YBX1* target site. YBX1 knock-down cells were transfected with pCS2 human *NODAL* cDNA with a 3 × MYC tag plasmid. After 24 h, the transfected cells were collected and lysed in RIPA buffer to extract proteins, and boiled for 3 min in Laemmli buffer. To assess changes in protein expression, western blot analysis was carried out with lysates from HEK293 cells that were un-transfected or transfected with human *NODAL* 3 × Myc alone, or YBX1 knock down plasmid together with human *NODAL* 3 × Myc. The primary antibodies used were: anti-c-Myc 1:5000 (Abcam32), anti-α-Tub1:5000 (Cell Signaling Technologies), anti-YBX1 1:5000 (Sigma).

## RESULTS

### Multiple Nodal pathway RNAs harbor a DLE-like element in their 3′UTRs

**Figure 1.**
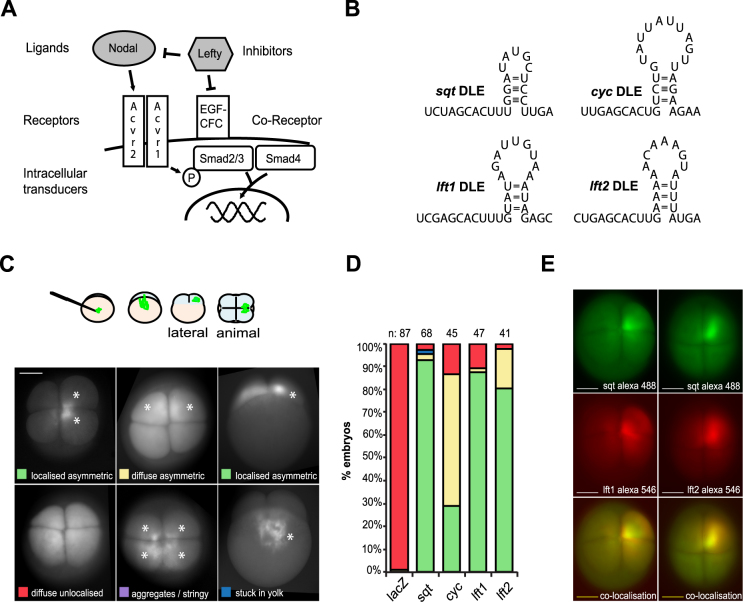
Multiple Nodal pathway component RNAs harbor a DLE-like element in the 3′UTR. (**A**) A simple schematic of the Nodal signaling pathway showing the Nodal ligands, the Activin receptors I and II (Acvr1/2), the Nodal co-receptor Epidermal growth factor/Cripto-1/FRL-1/Cryptic (EGF-CFC, one-eyed pinhead (Oep) in zebrafish), the intracellular signal transducers Smad2/Smad3 and Smad4, and the Lefty inhibitors of Nodal signaling. The grey highlighted components were tested for the presence of a dorsal localization element-like (DLE)/Y-box binding protein 1 (Ybx1) recognition module in their 3′UTRs. (**B**) Transcripts of the zebrafish nodals *nodal-related1/squint* (*ndr1/sqt*) and *nodal-related2/cyclops* (*ndr2/cyc*) genes, and the *lefty* inhibitors, *lft1* and *lft2*, harbor DLE-like motifs in the 3′UTR. The RNA motif is a composite of a short AGCAC sequence and a short hairpin/stem-loop. (**C**) Schematic showing the (fluorescent) RNA localization assay in zebrafish embryos. Fluorescent RNAs were injected into one-cell embryos, and RNA distribution was examined in four-cell stage embryos. The various categories for the distribution of the fluorescent RNA (localization) are shown in this representative set. The white asterisks point to the fluorescence signals from the injected RNAs. (**D**) Stacked column bar chart showing the proportion of embryos falling into different categories for localization after injection of fluorescent labeled RNA made from full length cDNA of different Nodal pathway components. A color code is used for the segments of the bar chart and the corresponding categories for localization. (**E**) Co-localization of injected *ndr1/sqt* RNA (green, Alexa 488) with *lefty1* and *lefty2* RNAs (red, Alexa 546) to the same cells in four-cell embryos. Scale bars in C and E, 0.2 mm.

Nodal and Nodal pathway components are regulated at the transcriptional and post-transcriptional level by similar control elements (31). To investigate if the DLE localization and translational control element we found in *sqt* is also present in other components of the pathway, we examined the 3′UTRs of Nodal pathway components. Transcripts for *nodal-related2/cyclops* (*ndr2/cyc*, hereafter *cyc*), *lefty1* (*lft1*) and *lefty2* (*lft2*) (18,32,33) contain motifs similar to the *sqt* DLE: an AGCAC sequence motif followed by a short hairpin (Figure 1A and B). To test the activity of these DLE motifs, we injected fluorescent-labeled RNA harboring the DLEs of *cyc, lft1* and *lft2* into fertilized zebrafish eggs, and examined if the reporter RNAs were transported and localized asymmetrically in one or two cells of the four-cell stage embryo (schematic Figure 1C, ‘localized asymmetric’). Fluorescent lacZ-βglobin UTR RNA served as a negative control. Fluorescent *lft1* and *lft2* RNA localized asymmetrically in the majority of injected embryos similar to *sqt* RNA. Injected *cyc* RNA is asymmetrically distributed, but not localized tightly in the majority of embryos (Figure 1D). If the *lefty* RNAs localize indeed similar to *sqt* RNA, then they should co-localize together with *sqt* RNA in localization assays. We co-injected differentially labeled *sqt* (green) and *lefty1* or *lefty2* (red) RNAs into one-cell stage embryos and found that they co-localized to the same cells in four-cell stage embryos (Figure 1E). These assays show that the *lft1* and *lft2* DLE motifs are similar in localization to the *sqt* DLE whereas the *cyc* DLE-like motif is somewhat divergent.

### A CA dinucleotide repeat masks the DLE in the *cyc* 3′UTR

**Figure 2.**
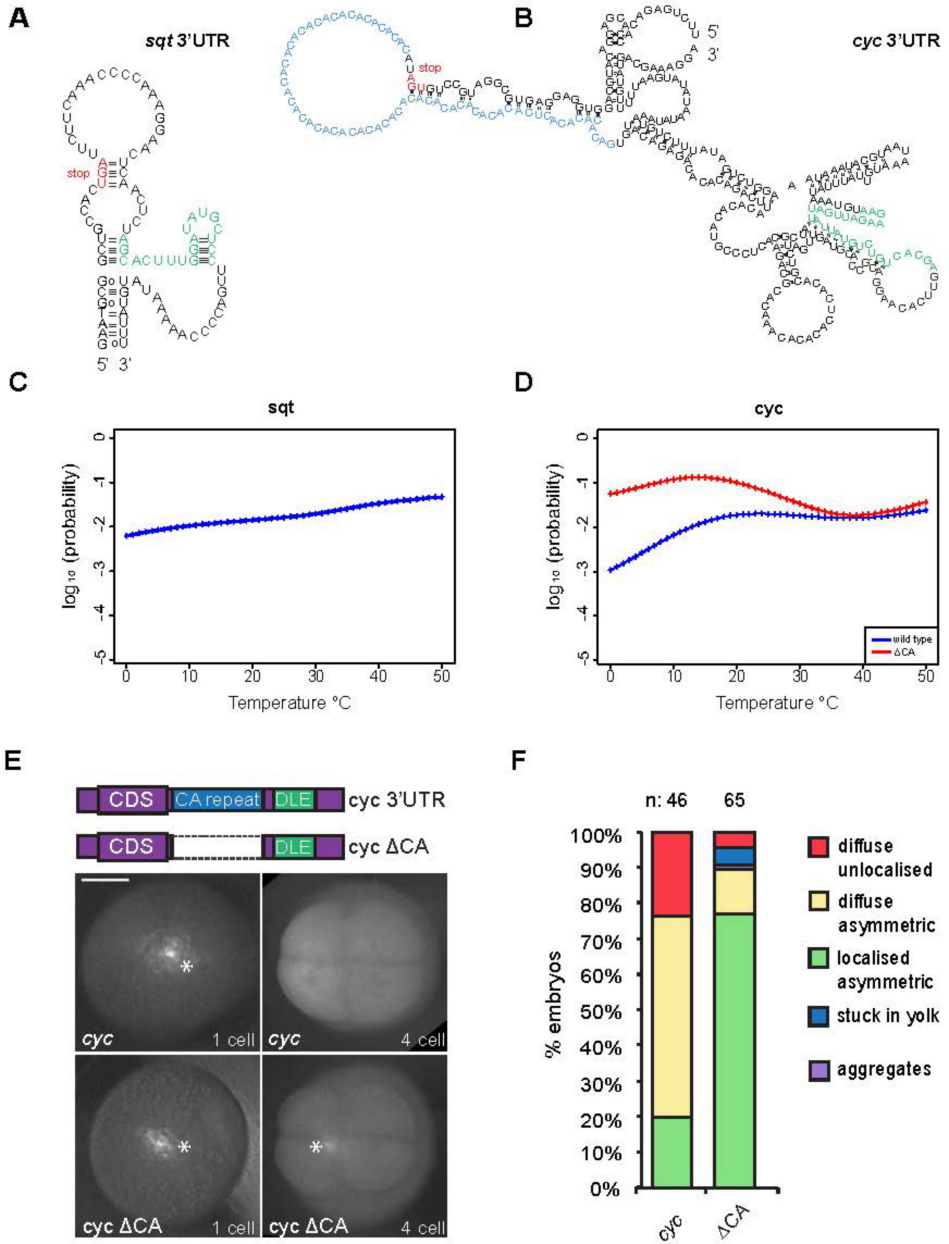
Activity of the DLE in *ndr2/cyc* is masked by a CA repeat in the 3′UTR (A,B) Modeled folding of the RNA sequence surrounding the *nodal-related1/squint* (*ndr1/sqt*, hereafter *sqt*) (**A**) and *nodal-related2/cyclops* (*ndr2/cyc*, hereafter *cyc*) (**B**) DLE elements. The *cyc* DLE (green) structure is distinct from the *sqt* DLE (green). A prominent CA-repeat in the *cyc* 3′UTR (light blue) is predicted to affect *cyc* RNA folding. Stop codons are shown in red. (C, D) Probability of *sqt* (**C**) and *cyc* RNA (**D**) to adopt a DLE structure (y-axis) at various temperatures (x-axis). The probability of a *cyc* sequence lacking the CA-repeat (*cyc*ΔCA, red) is higher than wild-type *cyc* RNA (blue) over a broad range of temperatures, including the temperature range in which zebrafish are commonly reared, 25–28°C. (**E**) Embryos injected with fluorescent *cyc* or *cyc*ΔCA RNA (see schematic of 3′UTR regions in top panel) show bright fluorescence in the middle of the embryo (white asterisks, left panels) immediately after injection. At the four-cell stage, *cyc* RNA is distributed diffusely whereas *cyc*ΔCA RNA localizes asymmetrically in most embryos (white asterisk bottom right panel). (**F**) Bar chart showing localization of *cyc* and *cyc*ΔCA RNAs in embryos. RNA distribution categories are shown on the right (boxes). Scale bar in E, 0.2 mm.

To investigate why *cyc* RNA activity is different from that of *sqt, lft1* and *lft2* RNAs, we first examined the secondary structure of the region encompassing the *cyc* DLE, and compared it to the secondary structure of the *sqt* DLE (Figure 2A and B). There is a tract of CA repeats near the *cyc* DLE and the overall structure of this 3′UTR is such that a DLE-like hairpin is not apparent in RNA structure predictions (Figure 2B and Supplementary material file 2). Computational analysis using RNAfold and RNAcop suggests that the probability for the formation of the DLE stem loop increases 2.3 to 3.1-fold and the over-all secondary structure of the *cyc* 3′UTR is predicted to be more stable when the CA repeat is deleted (Figure 2C and D and Supplementary material file 2) (22,34,35). To experimentally test this, we deleted the CA repeat and examined localization of fluorescently labeled *cyc* RNA lacking the CA repeat (hereafter called as *cyc*ΔCA) in embryos. Immediately after injection, both *cyc* and *cyc*ΔCA fluorescent RNAs are visible in the middle of the embryo. However, by the four-cell stage, *cyc* RNA is largely diffuse whereas *cyc*ΔCA RNA is localized in the majority of embryos (Figure 2E and F). Remarkably, deletion of the CA repeat from the *cyc* 3′UTR restores localization to near-*sqt* levels (Figures 1D and 2F). These findings suggest that although the DLE is present in *cyc* RNA, the CA repeat in the *cyc* 3′UTR masks and/or interferes with DLE activity in embryos.

### The *lft1* and *lft2* DLEs are similar to the *sqt* DLE

**Figure 3.**
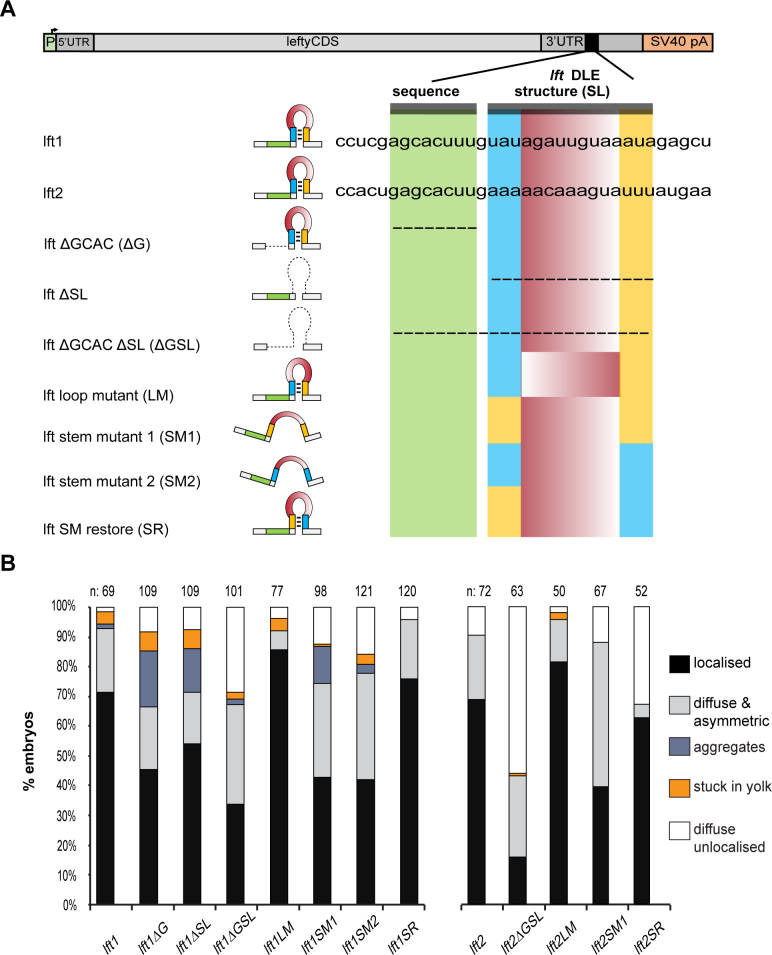
Features of the *lefty1* and *lefty2* DLEs critical for *in vivo* function (**A**) Schematic of the *lefty1* (*lft1*) and *lefty2* (*lft2*) DLE mutants tested in RNA localization assays. The top panel shows a schematic of the *lefty* constructs. The DLE is indicated by a black segment within the 3′UTR, and mutations in the *lft1/2* DLEs, their predicted structure and sequence are shown below. The AGCAC sequence motif region is highlighted in green, the yellow and blue bars indicate the stem regions, and the loop is highlighted in red. (**B**) Stacked bar chart showing the effect of the mutant *lft1* and *lft2* DLEs on RNA localization. The number of embryos scored is indicated above the bars. Colored boxes on the right show the various RNA distribution categories.

Fluorescent *lft1* RNA localizes similarly to *sqt* RNA in four-cell stage embryos. Efficient localization of *sqt* RNA requires the DLE, and the critical features required for *sqt* DLE function were previously identified by mutational analysis (15). To characterize the *lft1* DLE we generated a series of deletion and sequence-specific mutants affecting the *lft1* DLE and tested their activity by RNA localization assays in zebrafish embryos (Figure 3) The *lft1* DLE closely resembles the *sqt* DLE (Figure 1B). Deletions of either the sequence motif (ΔG) or the stem–loop structural motif (ΔSL) leads to mis-localized *lft1* RNA, and the frequency of mis-localization is increased upon deletion of both motifs (ΔGSL) (Figure 3A and B). Mutating the loop sequence (LM) does not seem to affect localization of *lft1* RNA, whereas mutations disrupting the stem (Stem Mutant or SM1/2) reduce localization to a similar extent as that seen with ΔGSL. Compensatory mutations that create a stem–loop even with a different sequence, e.g., by swapping the arms of the stem (Stem Restore or SR; Figure 3) can restore localization. We then carried out mutational analysis of the *lft2* and tested a set of sequence and deletion mutants in localization assays in 4-cell embryos as performed with *sqt* and *lft1* RNAs, and found that the *lft2* DLE behaves similarly to *lft1* and the *sqt/ndr1* DLE (Figure 3B). These results demonstrate that the *lft1* and *lft2* DLEs are composed of an AGCAC sequence motif adjacent to a short hairpin structure, which is similar to the *sqt* DLE.

### The *lft1* and *lft2* DLE elements are recognized by Ybx1

**Figure 4.**
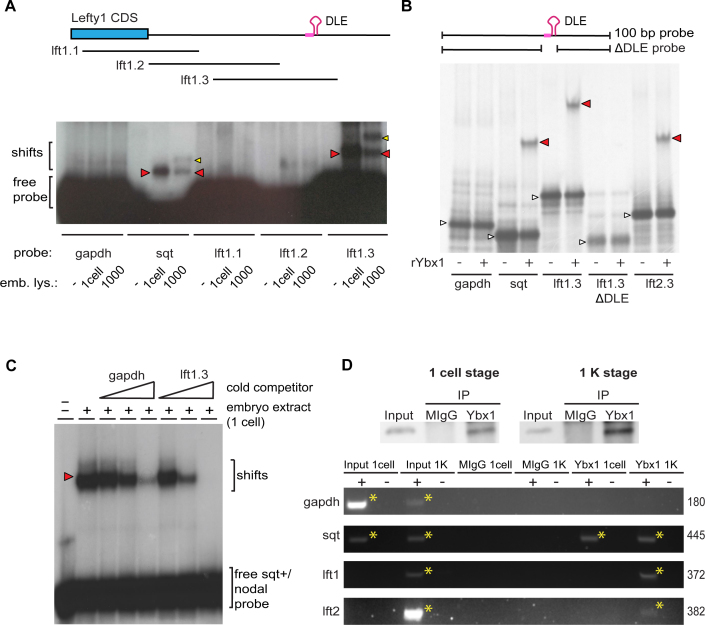
The *lefty1* and *lefty2* DLEs are bound by Ybx1. Electrophoretic mobility shift assays (**A**–**C**) and RNA-immunoprecipitation followed by RT-PCR (**D**) show binding of *lefty1* and *lefty2* RNAs to Ybx1 *in vitro* (A-C) and *in vivo* (D). (A) The schematic in the top shows the last coding exon of *lefty1* (*lft1* CDS, thick blue bar) and the 3′UTR (thin blue bar). The position of three different radiolabeled gel-shift probes lft1.1, lft1.2 and lft1.3, is shown. The *lft1* DLE is indicated in magenta. The autoradiogram shows gel mobility shifts of *nodal-related1/squint* (*ndr1/sqt*, hereafter *sqt*) and *lft1* probes incubated with lysates from 1 cell or 1000 cell stage embryos. Similar to radiolabeled *sqt* probe, mobility of the lft1.3 probe is shifted by embryo lysates (red and yellow arrow heads), in contrast to lft1.1 and lft1.2 probes, and control *gapdh* probe. The second band observed for the shifts of *sqt* and lft1.3 probes at the 1000 cell stage (yellow arrow heads) suggests binding of larger Ybx1-containing complexes to the probes at that stage, owing to modifications of Ybx1 itself, or due to binding of additional proteins. (B) Autoradiograph of an EMSA experiment testing the binding of radiolabeled RNA probes to recombinant zebrafish Ybx1 protein (rYbx1). The *squint*, lft1.3 and lft2.3 probes, which all carry a DLE, are shifted by recombinant Ybx1 protein (red arrow heads), but not the negative control *gapdh* probe and a lft1.3 probe lacking the DLE (lft1.3 ΔDLE). The white arrow heads point to unbound probe. (C) Autoradiograph of a gel loaded with *sqt* probe after incubation with embryo extract from 1 cell stage embryos and different concentrations of cold *lft1* (lft1.3) or *gapdh* probes. Red arrowhead indicates gel mobility shift. The first lane only contains the *sqt* probe (-). Closed triangles indicate progressive 4-fold increases in cold competitor. The *lft1* probe competes effectively with *sqt* for binding to Ybx1 compared to control *gapdh*, for which 4-fold more competitor is required. (D) RNA-immunoprecipitation from 1-cell and 1000-cell embryo lysates with anti-Ybx1 antibodies followed by RT-PCR. Western blots (upper panel) with anti-Ybx1 antibodies detect expression (input) and pull-down of Ybx1 in 1-cell and 1000-cell lysates but not with control mouse IgG antibodies. RT-PCR (lower panel) showing detection of *sqt* RNA in cDNA synthesized from anti-Ybx1 pull-downs from 1-cell embryos and from 1000-cell embryos, but not with control IgG pull-downs. PCR products for *lefty1* and *lefty2* are detected in RNA-IPs with anti-Ybx1 at 1000-cell but not in 1-cell lysates. Negative control *gapdh* is not detected in pull-downs at either stage. RT– controls show no detectable band and all inputs show the expected products.

The *sqt* RNA is bound via the DLE and translationally repressed by the nucleic acid binding protein Ybx1 to ensure that Ndr1/Sqt signaling is shut off in early zebrafish embryos. Because the *lefty* 3′UTRs behave similarly to *sqt* DLE in embryonic localization assays, we determined if the *lefty* DLEs are also bound by Ybx1. We tested if short (100 nucleotide) radiolabeled *lft1* 3′UTR probes are bound by factors in zebrafish embryo lysates in RNA gel-shift assays. Similar to a *sqt* 3′UTR probe harboring the DLE, a *lft1* 3′UTR probe (lft1.3) spanning the DLE is shifted by lysates from 1 cell stage and 1000 cell-stage embryos (Figure 4A). Such electrophoretic mobility shifts are not detected with the negative control *gapdh* probe or with *lft1* 3′UTR probes lacking the DLE (lft1.1 and lft1.2; Figure 4A and B). In gel-shifts using lysates from 1000-cell stage embryos, we observed an additional band with *sqt* and *lft1.3* probes (Figure 4A). This suggests binding of proteins of larger size to the probes at that stage, either owing to modifications of Ybx1 itself, or due to changes in the composition of the binding complex.

We then asked if the *lft1* and *lft2* 3′UTRs are recognized by recombinant Ybx1 using gel electrophoretic mobility shift assays. Short (100 nucleotide) 3′UTR radio-active labeled probes spanning the DLE regions in the *sqt, lft1* and *lft2* 3′UTRs (lft1.3 and lft2.3; Figure 4B) are shifted in the presence of recombinant zebrafish Ybx1 protein (rYbx1; red arrowheads in Figure 4B), whereas a gapdh control probe does not show a mobility shift with rYbx1 protein. In addition, we find no detectable shift with a lft1.3 probe lacking the DLE region (lft1 ΔDLE; schematic in Figure 4B). Thus, similar to the *sqt* 3′UTR, the *lefty1* and *lefty2* 3′UTRs are bound by recombinant Ybx1 and this binding requires the DLE region (Figure 4B).

We then tested the ability of unlabeled *lft1* probe to compete with radiolabeled *sqt* probe for binding to factors (Ybx1) from 1-cell stage embryo lysates (Figure 4C). Notably, *lft1.3* RNA is more potent as a competitor for nodal/*sqt* binding to rYbx1 compared to the *gapdh* control (Figure 4C). Thus, the RNA binding assays show that the *lft1* DLE is bound by Ybx1, and can compete with the *sqt* DLE for binding.

To determine if Ybx1 is in a protein-RNA complex with *lefty1* and *lefty2* RNAs *in vivo*, we performed RNA-immunoprecipitation (RNA-IP) with embryo lysates from 1-cell or 1000-cell embryos, followed by RT-PCR to detect *lft1* and *lft2* (schematic in Supplementary material file 2, Figure S6). Western blot analysis using anti-Ybx1 antibodies detect Ybx1 protein in pull-downs with 1-cell and 1000-cell stage lysates, in contrast to a negative control anti-mouse IgG antibody (Figure 4D, top panel). RNA-IP with anti-Ybx1 antibodies shows *lft1* and *lft2* RT-PCR products in 1000-cell stage lysates but not with 1-cell lysates, consistent with the known zygotic expression of *lft1* and *lft2* transcripts in embryos, whereas negative control (RT–) and RNA-IP using mouse IgG antibodies do not show any amplification product (Figure 4D). Conversely, we detected a *sqt* PCR fragment in RNA-IPs from 1-cell and 1000 cell stage embryos, as expected (Figure 4D and Kumari *et al.*, 2013), as also with input (positive control) from both stages (Figure 4D). Together, these experiments show that Ybx1 specifically binds to *lft1* and *lft2* RNA *in vitro* and in early zebrafish embryos.

### Ybx1 and DLE comprise a translational repression module for regulation of *lft1* and *lft2*

**Figure 5.**
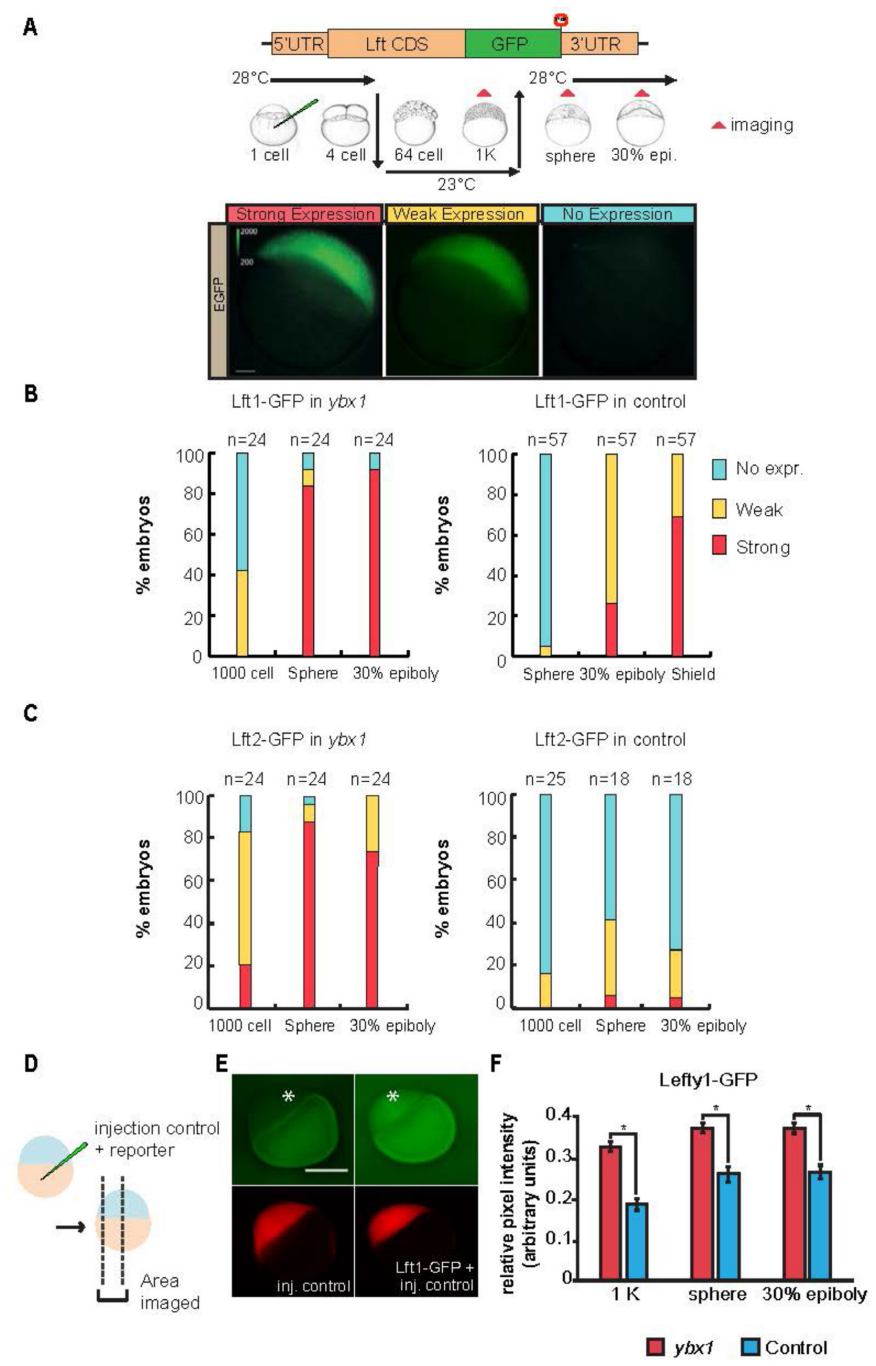
Ybx1 represses *lefty1* and *lefty2* translation. (**A**) Schematic of *lefty-gfp* translation reporters (top panel) indicating the location of GFP sequences (green box) and stop codon (red octagon), and outline of the temperature shift experiments (middle panel) to block maternal Ybx1 function using the temperature sensitive *ybx1*^sa42^ mutant. Embryos were imaged at three stages (red triangles): 1000 cell stage (1 K), sphere, and 30% epiboly. Bottom panel, representative embryos showing strong, weak or no GFP expression after injection of *lefty1-gfp* or *lefty2-gfp* mRNA (B, C) Stacked bar charts showing the percentage of Lefty1-GFP (**B**) or Lefty2-GFP (**C**) expressing embryos in each category (y-axis) at three different time points (x-axis). Categories are indicted by red (strong), yellow (weak) and blue (no detected expression) and number of embryos scored for each stage are provided. Lefty1-GFP and Lefty2-GFP expression is detected earlier in maternal *ybx1* mutant embryos compared to control (paternal *ybx1*) embryos. Furthermore, more embryos show strong expression of the reporters in *ybx1* mutants. (**D**–**F**) Quantitative measurement of GFP fluorescence in a defined region (black dotted lines in embryo schematic in D) of *lefty1-gfp* injected embryos (top panel in E) relative to an injected control (E, bottom panel) shows higher fluorescence intensity in maternal *ybx1* mutant embryos compared to control embryos. (F) Bar chart comparing relative pixel intensity of Lft1-GFP (y-axis) between maternal *ybx1* mutants (red bar) and controls (blue bar) at three different stages (x-axis) after injection of *lft1-gfp* RNA. Lft1-GFP is expressed at higher levels in *ybx1* mutants at all three stages. *N* = 34, error bars = SEM, asterisks indicate statistical significance at 0.05 by two-way ANOVA followed by Bonferroni *post hoc* test. Scale bar in A, 100 μm; lateral views of 30% epiboly embryos. Scale bar in E, 0.2 mm; embryo drawings in A from (76).

If the *lft1* and *lft2* DLEs are indeed regulated by Ybx1, then disruption of Ybx1 function should lead to mis-regulation of *lft1* and *lft2* translation. To test this possibility, we examined the expression of *lft1-gfp* and *lft2-gfp* translation reporters in zebrafish *ybx1* mutant embryos. Qualitative analysis of GFP expression (i.e. no expression, weak expression or strong expression) showed precocious and elevated expression of Lft1-GFP (Figure 5B) and Lft2-GFP (Figure 5C) in *ybx1* mutant embryos compared to control embryos. A significant proportion of *ybx1* embryos injected with *lft1-gfp* (10/24, 41.6%) show weak GFP expression starting from the 1000 cell stage (equivalent to 3 hpf) (Figure 5B). In control embryos, GFP expression is only observed from the sphere stage (4.3 hpf) (3/57, 5.2%) (Figure 5B) when the majority of *ybx1* mutant embryos show strong GFP expression (20/24, 83.3%). Even at late blastula / early gastrula stages (5 hpf, 30% epiboly) only a quarter of control embryos show strong GFP expression (15/57, 26.3%), whereas most *ybx1* mutants manifest very strong Lefty reporter expression. Similarly, in embryos injected with *lft2-gfp*, we observed strong GFP expression even at the 1000 cell stage in *ybx1* mutant embryos but not in controls, and the number of embryos expressing GFP increases in *ybx1* mutants at sphere and epiboly stages (Figure 5C).

We also performed quantitative analysis of GFP fluorescence intensity relative to an injection control (Figure 5D–F and Figure S7 in Supplementary material file 2). These analyses also show a clear increase in Lefty1-GFP fluorescence pixel intensity in *ybx1* mutant embryos compared to control embryos at all stages examined (Figure 5F and Supplementary Figure S7). Taken together, these findings show that Ybx1 is a translational repressor of *lft1* and *lft2* in zebrafish embryos.

**Figure 6.**
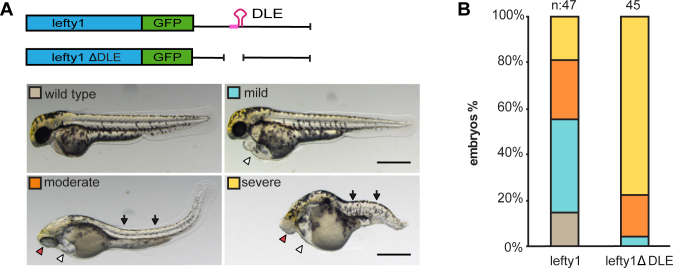
Deletion of the DLE in *lefty1* leads to higher *in vivo* Lefty activity. (**A**) Top panel, schematic representation of injected *lefty1-gfp* or *lefty1-gfpΔDLE* RNAs into wild-type embryos. Bottom, exemplar images of injected embryos showing the various loss-of-Nodal signaling phenotypes at 2 days post fertilization (wild-type, mild, moderate or severe). White arrowheads indicate cardiac edema, red arrowheads indicate cyclopia, and black arrowheads show midline defects in the trunk and tail. (**B**) Lefty1-Δ DLE causes a higher proportion of embryos with severe loss-of-Nodal signaling phenotypes. Scale bar 0.2 mm; lateral views with anterior to the left.

To determine if repression of *lefty1* is mediated through the DLE, we injected *lefty1-gfp* or *lefty1-ΔDLE-gfp* RNA into wild type embryos. In our assays, we did not detect an obvious difference in GFP fluorescence intensity in embryos injected with the two RNAs at equivalent doses (data not shown). However, analysis of embryo morphology at later stages shows that *lefty1-ΔDLE-gfp* injected embryos manifest more severe phenotypes compared to embryos injected with *lefty1-gfp* RNA (Figure 6A and B). The embryos displayed typical loss-of-nodal phenotypes observed upon *lefty1* overexpression: shortening of axis with reduction or lack of head and trunk mesendodermal derivatives including the heart, and midline defects including cyclopia, that were reported previously (32,33,36,37). The severe loss-of-Nodal phenotypes suggest higher activity of *lefty1-ΔDLE-gfp* than *lefty1-gfp*, leading to stronger inhibition of Nodal signaling. These phenotypes were also observed in compound *ybx1,sqt* mutants where zygotic Nodal signaling is reduced in the context of embryos lacking the DLE-binder, Ybx1 (17). Therefore, the DLE region in *lefty1* likely regulates Lefty activity in embryos through Ybx1. Taken together, these results show that Ybx1 and DLE comprise a regulatory module that represses *lft1* and *lft2* translation and activity in zebrafish embryos.

### Regulation of NODAL by YBX1 is conserved in humans

**Figure 7.**
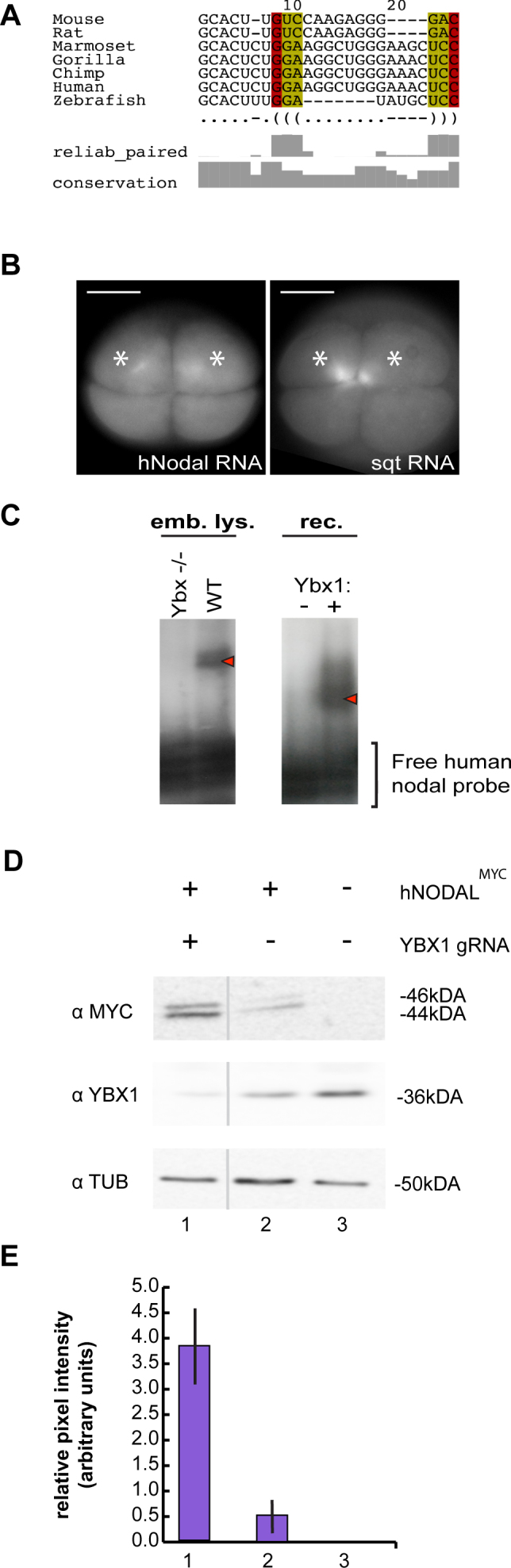
YBX1 regulates human NODAL protein expression. (**A**) conserved DLE-like motif in *sqt/ndr1* orthologues in dot-bracket notation showing sequence conservation (‘conservation’) and the reliability for conserved base pairs (‘reliab_paired’) based on PETfold predictions. (**B**) Comparison of the distribution of microinjected *nodal-related1/squint (ndr1/sqt)* and human *NODAL* fluorescent RNAs in four-cell stage zebrafish embryos. Similar to zebrafish *sqt/ndr1*, human *NODAL* RNA is asymmetrically localized with dense foci in two out of the four cells (white asterisks). (**C**) Autoradiograms showing RNA gel shift assays with human *NODAL (hNODAL)* radiolabeled probes. Shifts are observed with embryo extracts from wild type zebrafish embryos but not with embryo extracts from *ybx1* mutant embryos (red arrowhead, left gel). Mobility shift of hNODAL probe in the presence of recombinant (rec) zebrafish Ybx1 protein (right panel). (**D**) Western Blots of HEK cell lysates after transfections with myc-tagged human NODAL (hNODAL^MYC^) expression vector and YBX1 CRISPR/Cas9 targeting vector (YBX1 gRNA). The antibodies used for detection (left) and sizes (right) are indicated. Expression of processed and unprocessed hNODAL^MYC^ is increased in YBX1 knock-down cells. (**E**) Bar chart showing relative pixel intensity of NODAL^MYC^ expression normalized to α-Tubulin loading control. Standard error from three replicates is shown. Error bars = SD, asterisk indicates statistical significance at 0.05 by t-test. Scale bar in A, 0.2 mm.

NODAL and YBX1 are conserved between zebrafish and mammals including humans. We examined the NODAL 3′UTR in rodents and primates. Consensus structure predictions from multiple alignments of NODAL sequences in rodents and primates show the presence of a DLE-like element with a predicted stem region (red and yellow shading, Figure 7A) and loop region. In addition, human *NODAL* RNA localizes in 4-cell zebrafish embryos, similar to *sqt* and *lft1* DLEs (Figure 7B), raising the possibility that human NODAL might be regulated by the DLE/YBX1 module. To investigate if YBX1 regulates human *NODAL* RNA, we first tested if a human *NODAL* 3′UTR probe is bound by Ybx1. Recombinant zebrafish Ybx1 protein indeed binds to a human *NODAL* 3′UTR probe in gel shift assays (Figure 7C). A similar mobility shift of human *NODAL* 3′UTR probe is observed with wild-type zebrafish embryo extracts but not with *ybx1* mutant embryo extracts (Figure 7C). We then generated YBX1 mutant human HEK293T cells by CRISPR/Cas genome editing, and examined translation of a NODAL^myc^ reporter in YBX1 mutant cells (Figure 7D and E). Expression of human NODAL^myc^ (hNODAL^myc^) is significantly increased in YBX1^−/-^ cells compared to control cells (Figure 7D and E). These results show that similar to our findings in zebrafish, translation of human *NODAL* is also regulated by YBX1.

## DISCUSSION

Elucidating the mechanisms by which RNA elements are recognized by binding proteins and determining the dynamics of their interactions is crucial for comprehensive understanding of gene regulation (38). Computational prediction of RNA elements by sequence information alone has been confounded by many factors: (i) the sequences can be highly degenerate (ii) the elements might be structural motifs and (iii) the motifs might be a composite of sequence and structure. Moreover, some RBPs recognize RNA segments that might be very distant from each other in primary sequence, but are proximal in tertiary/quaternary structure. Advances in the computational prediction and experimental determination of RNA structure together with analysis of evolutionary couplings can facilitate the identification of RNA elements (39). Structure prediction can be improved, for instance, by co-variance analysis (40,41). Methods such as dimethyl sulfate treatment followed by deep sequencing (DMS-seq) and variants of selective 2′-hydroxyl acylation analysed by primer extension (SHAPE) allow the global, transcriptome-wide interrogation of RNA structures (42–46). Due to the current limitations in computational approaches, methods that allow precise interrogation and the experimental elucidation of RNA/protein interactions are indispensable tools for RBP target identification. The currently available methods are based on pull-downs of (tagged) RNAs and identification of the associated proteins or conversely, using antibodies to pull-down RBPs and identifying their bound RNA targets (47,48). However, these approaches do not provide functional evidence *in vivo* in the physiological context.

We determined the sequence/structure requirements for the functionality of an RNA element, the DLE, and tested if similar RNA elements in other components of the same pathway had the same requirements. Predictions for minimum free energy (MFE) RNA secondary structures do not strictly correspond to the *in vivo* functional structure and have limited accuracy, especially for larger RNAs (49). Therefore, the predicted structures (Supplemental material file 2) are potential RNA structures while other structures with a similar thermodynamic stability may constitute native structures. The activity of the predicted DLEs was derived from functional localization assays in live zebrafish embryos.

We have shown previously that *sqt/nodal* RNA localizes in eggs and early embryos (14,17,50,51). Although the other nodal pathway RNAs - *cyclops, lefty1*, and *lefty2* - are not expressed in 4-cell embryos, three out of four 3′UTR elements could be validated *in vivo* in our tests of DLE function, where we used localization as a read-out of biological activity of the 3′UTRs. This is supported by our results showing binding of *lefty1* and *lefty2* DLEs to Ybx1 and elevated and premature translation in *ybx1* mutants. This illustrates the ease of using such RNA localization and translation assays in live zebrafish embryos and the potential of our approach for large-scale *in vivo* functional screens for RNA elements.

In contrast to most *in silico* methods for predicting RNA motifs including structures, our approach infers specific motif patterns from experimental observations, and these may not be fully captured by automated approaches. More parameters may need to be considered for improving current *in silico* prediction methods. Refinements in our analysis may further reduce false positive predictions and enhance the applicability of our approach for RNA motif discovery (52–54).

Remarkably, we also found that a simple sequence repeat insertion in the 3′UTR of *cyc* disrupts the activity of this DLE. This might be either because the overall RNA secondary structure folding is altered, and/or the binding of other proteins required for optimal DLE function is disrupted. Our findings indicate that the context of the DLE is crucial for biological activity. Similar requirements for local secondary RNA structure in recognition has been reported for the RBPs MBNL1 and RBFOX2 *in vitro* (55). The RNA recognition motif (RRM) is one of the best-studied RNA binding domains, and minimally binds to dinucleotides. Several RBPs, such as the RRM-containing RBPMS proteins bind to CA repeats (56). An alternative explanation for our observation that a CA repeat in the 3′UTR disrupts the function of the *cyc* DLE could be that binding of RBPs to the CA repeat *in vivo* might interfere with DLE function, by preventing binding of Ybx1 to the *cyc* DLE, or perhaps by destabilising *cyc* RNA.

The *cyclops* gene is structurally different from other genes in the Nodal pathway. In addition to the CA-repeat in its 3′UTR, the *cyc* locus harbours insertions and repeat elements (including many Proline-rich sequences) in the protein-coding region, that we reported previously (57). The Proline repeats in the coding region render Cyc/Ndr2 protein less stable and with a lower signaling range compared to Sqt/Ndr1 (57,58). Notably, the *cyc* locus is located close to the telomere on chromosome 12, a region that is known to be dense with repetitive elements (31,59). The nodal-related locus *sqt*, many *nodal* genes across species, and *lefty1* and *lefty2* do not harbor such repeat elements. Our findings suggest that the repeat insertions into the *cyc* gene have profound effects on its regulation.

We note that the DLE deletions in *lefty* RNAs substantially reduce localization, but do not abolish all DLE activity. We had observed this previously with the *sqt/nodal* 3′UTR as well (15). This suggests that although the DLE is the main region, sequences outside the DLE also contribute to activity of the *lefty* 3′UTRs. Alternatively, the residual localization activity of exogenous *lefty* RNAs lacking the DLE might arise from hitchhiking of localizing RNAs such as endogenous *sqt* RNA via RNA:RNA interactions. Such interactions have been found to enhance RNA localization in fly oocytes (60).

Our finding that both ligand and inhibitors in the Nodal signaling pathway are regulated by a DLE/Ybx1 module is noteworthy. Post-transcriptional regulation of the Nodal pathway through the small regulatory RNA miR-430 was reported to dampen and balance Nodal signaling by mRNA degradation (61). However, miR430 is not active at the earliest stages of zebrafish development, prior to its transcription from the zygotic genome (62). Maternally provided proteins deposited in the egg, such as Ybx1, control RNAs (including Nodal pathway transcripts) in early embryos prior to miR-430 function (63). Translational inhibition of transcripts carrying DLEs could, potentially, also arise from reduced mRNA stability, as shown for miR-430 mediated repression and mRNA decay (62,64). While enhanced decay through the DLE is in theory possible, we have shown previously that maternal *sqt*/nodal RNA levels are uniform in early zebrafish embryos (prior to ZGA) (see Supplemental Figure S1 in (14)). At these stages, Ybx1 represses translation of *sqt/nodal* (17). Therefore, it is unlikely that mRNA decay plays a role in DLE-mediated repression of *sqt/nodal* in early zebrafish embryos.

Interestingly, the minimal DLE we identified (in *lefty* and *nodal* RNAs) as responsive to Ybx1 is a short AGCAC sequence motif followed by a hairpin. The sequence and length of the loop differs considerably between *sqt, lft1* and *lft2*, and yet *in vivo*, these elements behave similarly. It therefore seems likely that the Ybx1-binding site comprises of the short sequence and stem region, and the variable loop region in the DLE likely does not contribute significantly towards Ybx1 recognition.

Work in organisms such as *C. elegans, Xenopus* and *Drosophila* has provided substantial insights into RNA localization elements. Amongst the best studied are a group of RNA localization elements that facilitate apical localization of RNAs in *Drosophila* embryos at blastoderm stages (65). The elements form secondary structures involving stem-loops. However, the complexity of the structures formed by the minimal elements ranges from a single stem-loop (*K10*) to over two stem-loops (*h*) to multiple stem loops (*bcd*) (8,9,66). A key feature identified through study of these elements is the importance that shape plays in the function of the elements. For example, the *Drosophila K10* transport and localization signal (TLS) contains A’-form helices important for its function (66). This demonstrates that besides secondary structure, RNA conformation is a key feature in RNA elements recognized by RBPs. Sequences that facilitate intermolecular (RNA:RNA) interactions and quaternary structure can enhance the function of RNA localization elements (60). Studies of *Drosophila* localization elements have demonstrated the importance of precise spacing and orientation of features within the elements for their function (67). The localiszation machinery seems to recognize the A’-form helices in *K10* RNA only when correctly oriented relative to each other (10). Similarly, the spacing between a bulge and a loop is critical for the recognition of TAR RNA by Tat protein (68). In our study, we show that the DLE is a composite sequence and structure motif that controls localization and translation of Nodal and its inhibitors. However, much remains to be known about precisely how the DLE functions. It is likely that identification and analysis of more DLE-like elements will reveal additional features that are important for its function.

Previous studies using position weight matrix analyses and *in vitro* binding with short oligonucleotides (SELEX) had predicted some YBX1 sites in mammalian cells (69,70). However, there is little overlap between the analyses and their probability scores are low. Importantly, some known YBX1 targets are not predicted, and neither NODAL nor LEFTY were found, suggesting that not all YBX1 targets were captured in these analyses. By contrast, through our *in vivo* assays for localization and translation and *in vitro* binding, we found evidence for the conservation of translational regulation of Nodal by a DLE/YBX1 module between zebrafish and humans. Thus, our strategy can be used more generally for *in vivo* validation of RNA elements.

Our assay is also useful for testing heterologous UTRs for activity in zebrafish, as we showed for human *NODal.* Human *NODAL* RNA localizes in early zebrafish embryos, albeit somewhat distinctly from zebrafish *sqt* RNA. Nonetheless, we observed dense clusters of fluorescent human *NODAL* reporter RNA in one or two cells of four-cell stage embryos, similar to zebrafish *sqt/nodal* and unlike control lacZ RNA or the various DLE mutant RNAs. Moreover, Ybx1 is very well conserved between zebrafish and humans. Human YBX1 and zebrafish Ybx1 proteins are composed of the same protein domains (17), with highly similar sequences: the cold shock domains (CSD) are 100% identical, the dimerization domains (DD) are 81% identical, the actin binding domains (ABD) are 63% identical, the single stranded DNA-binding domains (ssDBD) are 67% identical, and the nuclear localization sequences (NLS) are 65% identical. The overall identity between the two protein sequences is 72%. Unlike many other zebrafish genes, *ybx1* has no duplicate (ohnologue) in the zebrafish genome. Therefore, it is likely that the Ybx1 orthologues carry out similar, conserved functions in the regulation of gene expression. Accordingly, we demonstrated conservation of the regulation of human *NODAL* RNA by the DLE/YBX1 module between zebrafish and humans by showing: (i) the presence of a DLE in human *NODAL* RNA, (ii) similar behavior of human *NODAL* RNA to *sqt* RNA in RNA localization assays, (iii) binding of recombinant zebrafish Ybx1 to a *NODAL* 3′UTR probe in gel shift assay and (iv) elevated translation of a human *NODAL* translation reporter after CRISPR/Cas9 mediated depletion of YBX1 protein in human cells.

Human NODAL has been implicated in the maintenance of ESC cell pluripotency and in tumor metastasis. This raises the exciting possibility that this mechanism of translational control might regulate Nodal signaling in different developmental, physiological, and pathological contexts in humans, and warrants further exploration. Notably, human YBX1 has been found in mRNPs in neurons, a cell type known for extensive RNA transport, subcellular localization and localized translation (71–74). Furthermore, Ybx1 has been proposed as a facilitator of RNA transport/localization by recruiting RNAs to the cytoskeleton (16). Ybx1 has also been found to control splicing and translation of many mammalian RNAs. Therefore, it is conceivable that Ybx1 regulates other endogenous zebrafish RNAs, potentially in neurons. Our study demonstrates translational co-regulation of multiple Nodal pathway components by a shared RNA element. It is plausible that this (DLE/Ybx1) or similar modules are deployed to regulate sets of functionally related transcripts in a variety of contexts as part of RNA regulons (75).

## Supplementary Material

Supplementary Data
