# Supplementary material for: Translational co-regulation of a ligand and inhibitor by a conserved RNA element
Source: Nucleic Acids Res. 2017 Oct 20;46(1):104–19. doi: 10.1093/nar/gkx938 (PMC5758872; doi:10.1093/nar/gkx938)
Supplement: Supplementary Data [file gkx938_supp.pdf]

SUPPLEMENTARY MATERIALS

Supplementary Table S1. List of primers and sequences.

Supplementary Material File 2: RNA secondary structure analysis including supplemental tables S2-S4, Supplemental figures S1-S6, and Supplementary references.

Supplementary Table S1: Primer list

	Primer name	Sequence
		Primers for generation of constructs
1	wSDM1.lft1 dGCAC.F	ttactagatacctcggtatagattgtaaatagagctaattgatatgcg
2	wSDM1.lft1-dGCAC.R	atttacaatctataccgaggtatctagtaagggttaaattgacaacacc
3	wSDM2.lft1-dSL.F	acctcgagcactttggagctaattgatatgcgtttatttttgcatttg
4	wSDM2.lft1-dSL.R	catatcaattagctccaaagtgctcgaggtatctagtaaggg
5	wSDM3.lft1-dGCAC-dSL.F	ttactagatacctcggagctaattgatatgcgtttatttttgcatttg
6	wSDM3.lft1-dGCAC-dSL.R	catatcaattagctccgaggtatctagtaagggttaaattgacaacacc
7	lft1_SL1F	ACCTCGAGCACTTTGATAAGATTGTAAATAGAGCTAATTGATATGCG
8	lft1_SL1R	ATTTACAATCTTATCAAAGTGCTCGAGGTATCTAGTAAGGG
9	lft1_SL2F	CTTTGTATAGATTGTAATATGAGCTAATTGATAGTTTATTTTTG
10	lft1_SL2R	CGCATATCAATTAGCTCATATTACAATCTATACAAAGTGCTCGAGGTA
11	lft1_SL3F	ACCTCGAGCACTTTGATAAGATTGTAATATGAGCTAATTGATATGCGTTTATTTTTGCATTTG
12	lft1_SL3R	CATATCAATTAGCTCATATTACAATCTTATCAAAGTGCTCGAGGTATCTAGTAAG
13	Lft1 SL mut F	CGAGCACTTTGTATTCTAACATTATAG
14	Lft1 SL mut R	GCATATCAATTAGCTCTAAATGTTAGA
15	cyc fl cDNA F1 EcoRI	gtgGAATTCGCGCATCATGCACGCGCTCGGAG
16	cyc fl cDNA R1 SalI	gcgaGTCGACctattagcaaaaatatctccaatctaatcatcagatctcc
17	cyc dCA F1	agatgtacagagacacacagacttacacacatgccctc
18	cyc dCA R1	aTCAcaggcatccgcactcctc
19	pCS2+ -45F	CGTGCCTAATGGGAGGTC
20	pCS2+ 352R	GGCCCAATGCATTGGCGCCG
		Primers for lefty1 probe synthesis
21	lft1 -100.1 T3F	AATTAACCCTCACTAAAGGGAGATGATTGTGGAAAAATGTGGATG
22	lft1-100.1 R	aggtagtatagtgcgtcatgtag
23	lft1-100.2 T3F	AATTAACCCTCACTAAAGGGAGAatagcctgtgtgtatcgcccag
24	lft1-100.2 R	tatctagtaagggttaaattgac
25	lft1-100.3 T3F	AATTAACCCTCACTAAAGGGAGAaaagttataagatttagaatgg
26	lft1-100.3 R	taaacgcatatcaattagctcta
		Primers for RT-PCR
27	Gapdh_3'UTR_Fw	CTGACAGTCCGTCTTGAGAAAC
28	Gapdh_3'UTR_Rev	AGTGATCGTTGAGAGCAATACC
29	sqtFwExon2	GAAGGAACCACAGAACTGATGATA

30	sqtRevExon3	GAGCATATCCAAAGTGCTAGAGTT
31	Lft1 exon2 f	CTCTACAAGAAGGCCCCACA
32	lft1_Rev-exon3	CTCCTCTAGGTTGAGTGTGTAAAG
33	lft2 fw	TTCATTACTGGTCTAAATCCCAAAA
34	lft2 rev	CTCTGTCCATATCCATAGAAACCAC

Supplementary Material file 2: additional RNA secondary structure analysis

Analyzed sequences

Sequences
cyc/ndr2 wt (3'UTR + 50nt CDS)
**UUCUGAGACACCACGAGGACAUGCAGGUGGAGGAGUGCGGAUGCCUGUGA**CACACACACACACACACAC<br>ACACACACACACACACACACACACACACACACACACACACACUCACACACACAGAUGUACAGAGACACACAGACUU<br>ACACACAUGCCCUCACAGACGCACAAACACACACUCACACGUCUGCAGUUAUGAUGCACCGCAGGAACACUUG<br>AGCACUGUCUGUAUUAUUAGUUAGAAGAAUGUAAAUAUUUAUUGUAAAUAAUGCAUAAAUAAAGGUCUGAUA<br>UUUCUGUAAAUAUAAUAUAUGAAUUUUUGUAUGACGAAAGGAGAUCUGAUGAUUAGAUUGGAGAUAUUUUUG<br>CUAAUAG
cyc/ndr2 dCA (3'UTR + 120nt CDS)
**CCUGCAUCCUGCUGCGCUCCGACCCGCACCAGCGCUCUCAGUAUGCUGUACUACGAGAACGGAGAGAUGA**<br>**UUCUGAGACACCACGAGGACAUGCAGGUGGAGGAGUGCGGAUGCCUGUGA**UAGAUGUACAGAGACACACA<br>GACUUACACACAUGCCCUCACAGACGCACAAACACACACUCACACGUCUGCAGUUAUGAUGCACCGCAGGAAC<br>ACUUGAGCACUGUCUGUAUUAUUAGUUAGAAGAAUGUAAAUAUUUAUUGUAAAUAAUGCAUAAAUAAAGGUC<br>UGAUAUUUCUGUAAAUAUAAUAUAUGAAUUUUUGUAUGACGAAAGGAGAUCUGAUGAUUAGAUUGGAGAUAU<br>UUUUGCUAAUAG
sqt/ndr1 (3'UTR + 50nt CDS)
**UCAUGAGACACCAUGAAGGCAUGGUUGUUGCAGAAUGCGGCUGCCACUGA**UUCUUCAAACCCCAAAGGAAC<br>UCAACUCUAGCACUUUGGAUAUGCUCCUUGACCCCAAAAAUAUGUAUUUAAGAAAAACUGCUGUCAAUUAUUC<br>CCACUUGAAAUUAUUAUGGUUUCCUGCACUGAGGCACCUGGAUAACUUGAUGCUAUUAUUGAAAGCUUUGCGU<br>GUUUGCCUUAUCUGUAAAUAGUAGAGUAUGUAAAUUACCAAAUG
lefty1 (3'UTR + 50nt CDS)
**UGAUUGUGGAAAAAUGUGGAUGCGCAAUGGACAAUAUUUCAGUUGUAUAA**AUAGCCUGUGUGUAUCGCCCA<br>GCUAUCUACAUGACGCACUAUACUACCUAAAGUUAUAAGAUUUAGAAUGGGUGUUGUCAAUUUAACCCUUACU<br>AGAUACCUCGAGCACUUUGUAUAGAUUGUAAAUAGAGCUAAUUGAUAUGCGUUUAUUUUUGCAUUUGUAUAU<br>GAUGCCAUUGAUUUGUAGAUGUUUUAUUUUGAGCAUAUAGCACGGGUUUGUUGUAUAUGUGAGACUGACU
lefty2 (3'UTR + 50nt CDS)
**UGAUUGUGGAGAAGUGUGGAUGCUCCAUGGACAAAAUCCCUCCAGUCUAA**CAGUGUGGUGUCGAAUAGUUU<br>GCUCUCAGAUGAGAGGUGACGUGUUUAAUAAUCUCAGUUAUGGGACUCCCCAAAUCCCCACUGAGCACUUGAA<br>AAACAAAGUAUUUAUGAAUUAUUGCUUUAUGUAAUGUAAGCUUUAGAAAUGGCUUGUUGAGAGAUGUGAUGU<br>UUGAAAAAUAAAGUAAGUAGCU

Table S2: Analyzed sequences. The analyzed sequences contain the 3'UTR and 50nt of the CDS. For cyc, we extended the wild type cyc UTR sequence 50nt upstream into the CDS and the cyc sequence with the deleted CA repeat (dCA) 120nt upstream into the CDS to match their overall sequence lengths for the sake of comparability. The sequence of the CDS is highlighted in bold.

Constrained folding

To investigate the presence of a potential RNA secondary structure inside the cyc UTR that folds into the assumed DLE stem loop, we used RNAfold for constrained folding (1). Here, we constrained only base pairs of the DLE stem loop and left all other nucleotide flexible. Predictions were performed at a temperature of 25 °C using energy parameters refined by Andronescu et al. (2) . We also performed classic minimum free energy (MFE) structure predictions with RNAfold, i.e. folding without constraints, to compare differences in free energy (Table S4). Compared with the wild type sequence, MFE structures predicted for the cyc dCA sequence exhibit a substantially lower free energy (> 25 kcal/mol difference) indicating an increased stability for overall RNA secondary structure formation. MFE structure predictions with constrained DLE stem loops exhibit similar but slightly increased

free energies (< 2 kcal/mol). These results suggest that the formation of the assumed DLE stem loop allows folding into a RNA secondary structure with similar thermodynamic stability as a the MFE structure prediction without any constraints. See figure S1-S5 for RNA secondary structure diagrams.

Structures in dot bracket notation
cyc MFE wt
..(((.....((....)).....)))(((...(((((.(..((..((((..)))).)).).))))).))).(((((((.((((((((....(((((((((.((((((..((((((((((.................))))))..(((....(((...)))..)))...)))))).))).))))((((..(((.....))))))).(((((((.((.......))))))))..))))))...)))))))).((((...))))...(((((((...)))))))...))))).))(((((...(((....)))...))))))..
cyc MFE dCA
..((((((..(((((.(((((...(((....))).((((((((...)))))..)))).)))))((((....((((.......((((.((((...(((...((((((....))(((((.....(((......))))))))))).))).)))).))))......(........)...))))..))))))))))...))))))..((((..(((.........)))))))..((((((...((((((((...(((((((.((.......)))))))))..(((((....((((.((..((((((...........)))))).))))))..)))))((((......))))...)))))))))))))).
cyc MFE wt constrained stem loop
..(((.....((....)).....)))(((...(((((.(..((..((((..)))).)).).))))).))).(((((((.((((((((....(((((((((..(((((..((((((((((.................))))))..(((....(((...)))..)))...)))))).))((((..........))))....))))).(((((((.((.......))))))))...)))))...)))))))).((((...))))...(((((((...)))))))...))))).))(((((...(((....)))...))))))..
cyc MFE dCA constrained stem loop
..((((((..(((((.(((((...(((....))).((((((((...)))))..)))).)))))((((....((((.......((((.((((...(((...((((((....))(((((.....(((......))))))))))).))).)))).))))......(........)...))))..))))))))))...))))))..((((((...(((..(((((.((((..........))))))..)))...(((((((.((.......))))))))))).)).((((((((((..((((((...........)))))).))).......(((((((...))))))))))))))).))))......

Table S3: Predicted structures for the cyc UTR using constrained folding. Predictions are based on the sequences given in Table S2 at 25 °C.

	Free energy in kcal/mol
cyc MFE wt	-89.02
cyc MFE dCA	-116.11
cyc MFE wt constrained stem loop	-88.09
cyc MFE dCA constrained stem loop	-114.80

Table S4: Free energy of predicted RNA structures. Structure predictions are based on sequences in Table S2. Table S3 shows the predicted structures at 25 °C.

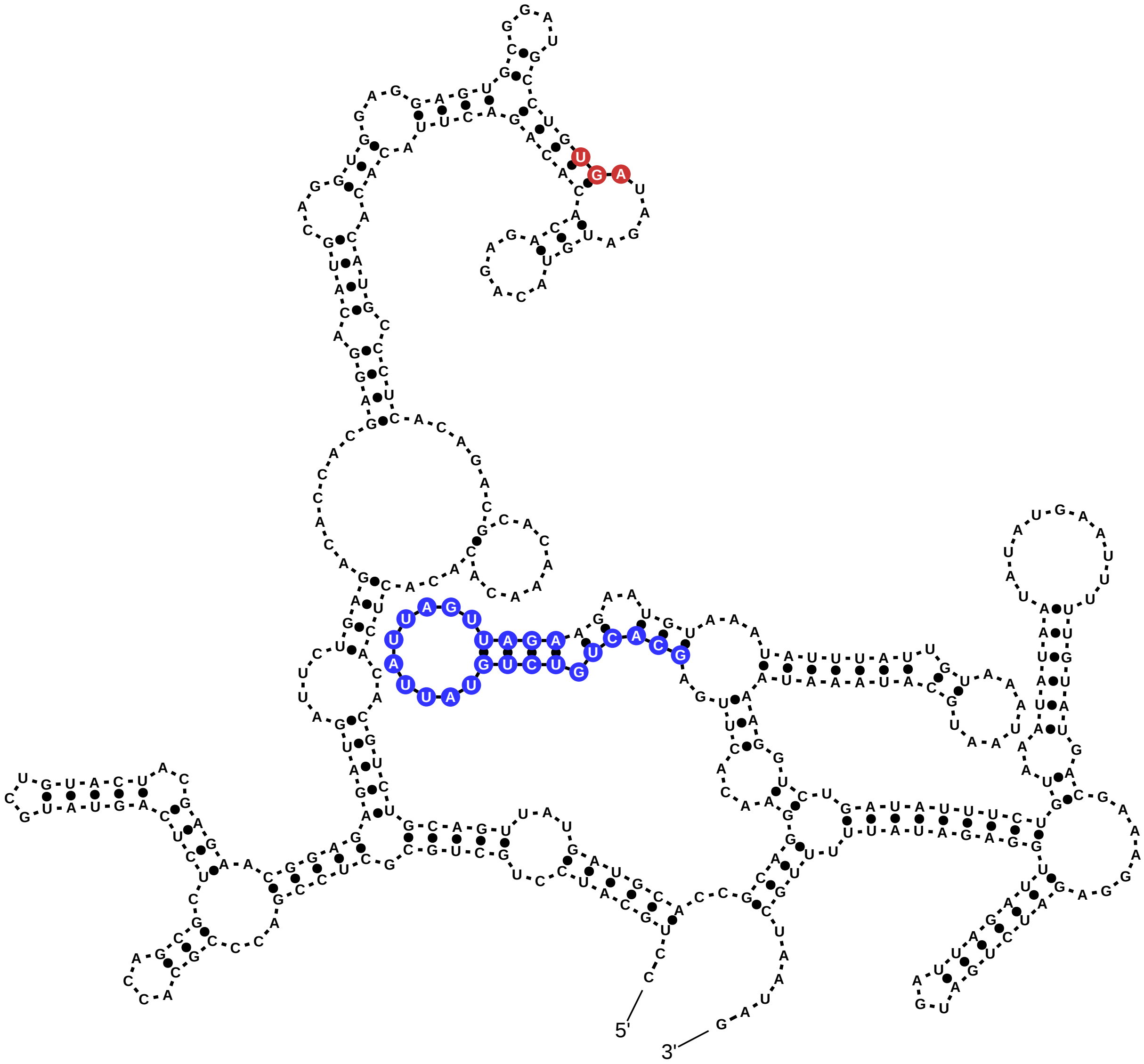

Figure S1: Secondary structure diagram of the dCA sequence MFE structure with constrained DLE stem loop at 25 °C. Blue circles indicate the DLE motif. Red circles show the stop of the CDS. The corresponding sequence is given in Table S2.

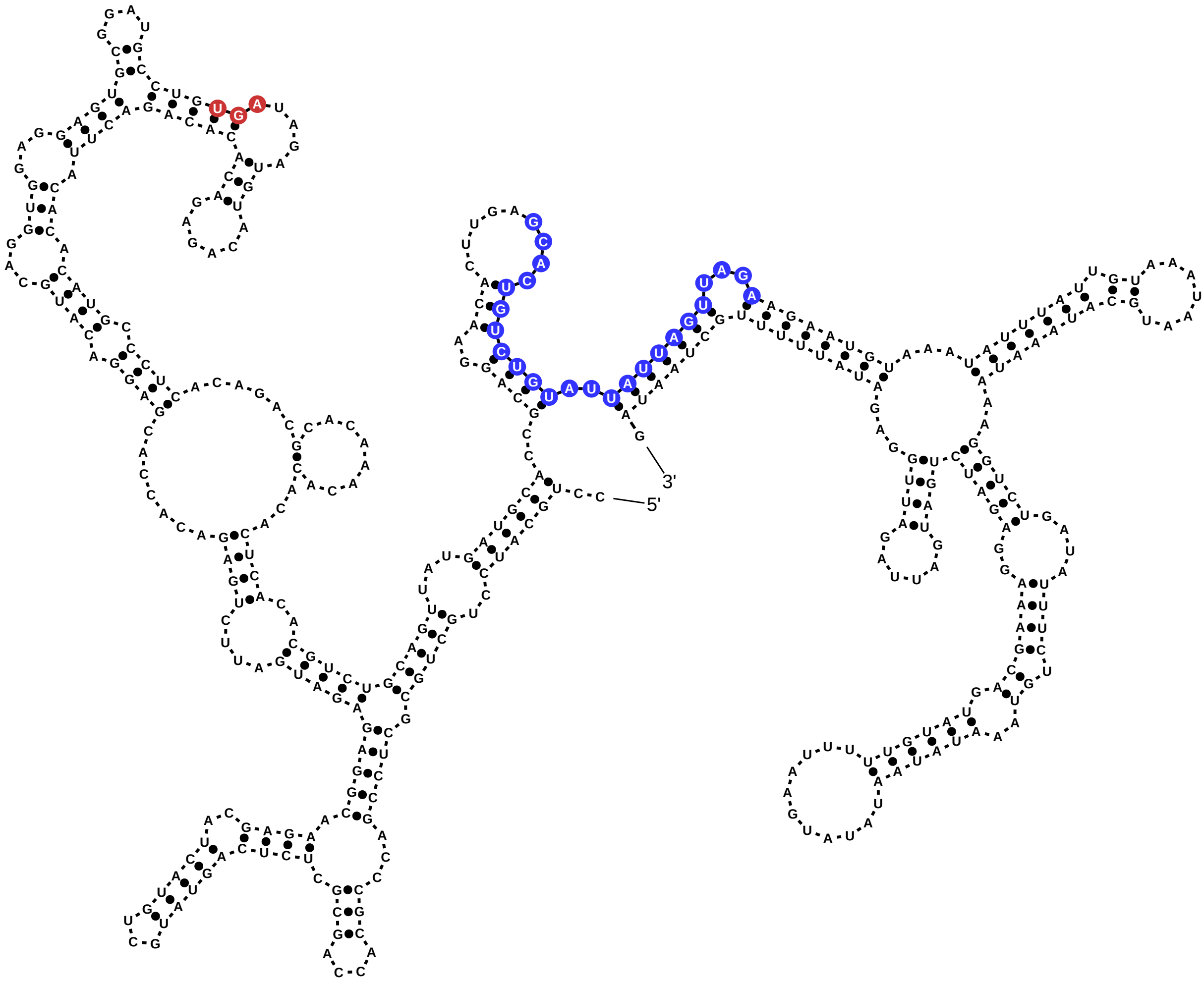

Figure S2: Secondary structure diagram of the dCA sequence MFE structure at 25 °C. Blue circles indicate the DLE motif. Red circles show the stop of the CDS. The corresponding sequence is given in Table S2.

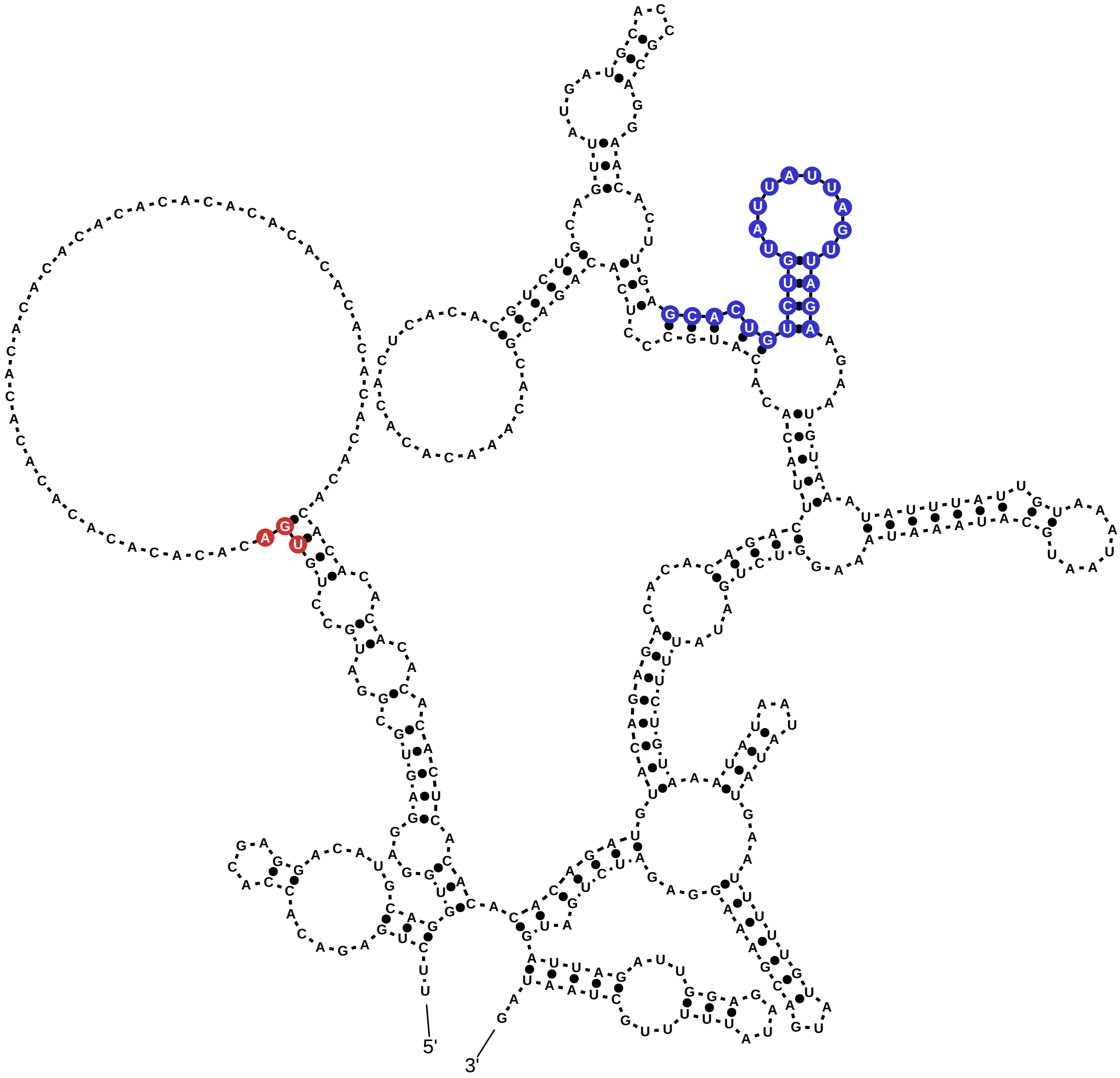

Figure S3: Secondary structure diagram of the wild type MFE structure with constrained DLE stem loop at 25 °C. Blue circles indicate the DLE motif. Red circles show the stop of the CDS. The corresponding sequence is given in Table S2.

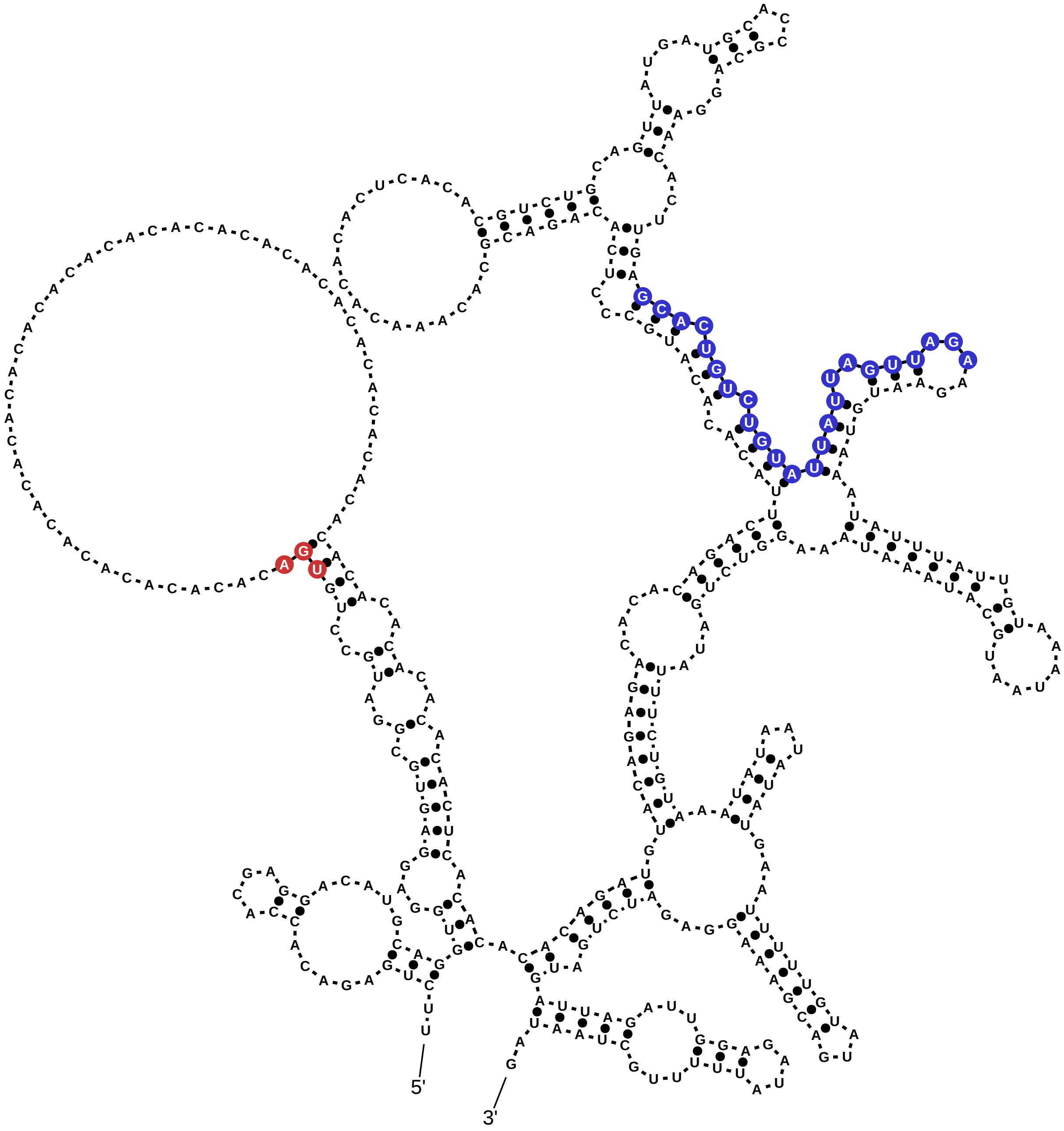

Figure S4: Secondary structure diagram of the wild type MFE structure at 25 °C. Blue circles indicate the DLE motif. Red circles show the stop of the CDS. The corresponding sequence is given in Table S2.

Temperature dependence of DLE stem loop formation

Since we were in particular interested in analyzing the formation of the DLE stem loop, we compared the probabilities for folding into the stem loop using RNAcop (3). RNAcop computes the probability for folding into a constrained sub structure while considering all possible RNA secondary structures and different flanking region lengths. Similarly to the constrained folding mentioned above, we only constrained base pairs of the DLE stem loop, left all other nucleotides flexible and used the refined energy parameters by Andronescu et al. (2). In the following, we show results where all nucleotides from the input sequence up- and downstream of the DLE stem loop constitute the flanking regions (Table S2). We computed the probability for folding into the DLE stem loop for temperatures in a range from 0 °C to 50 °C (Figure S5). Note that the probabilities for DLE stem loop formation might not be directly comparably due to different UTR lengths of the transcripts. For lefty1 and lefty2, there is a clear increase for the probability to fold into the DLE stem loop when temperatures are increased whereas the increase in probability is much less pronounced for sqt. For cyc, the deletion of the CA repeat sequence results in a clear increase in probability for DLE stem loop formation for temperatures below approximately 35 °C. For instance, we predicted a 3.8-fold increase in probability for the DLE stem loop formation at 23 °C.

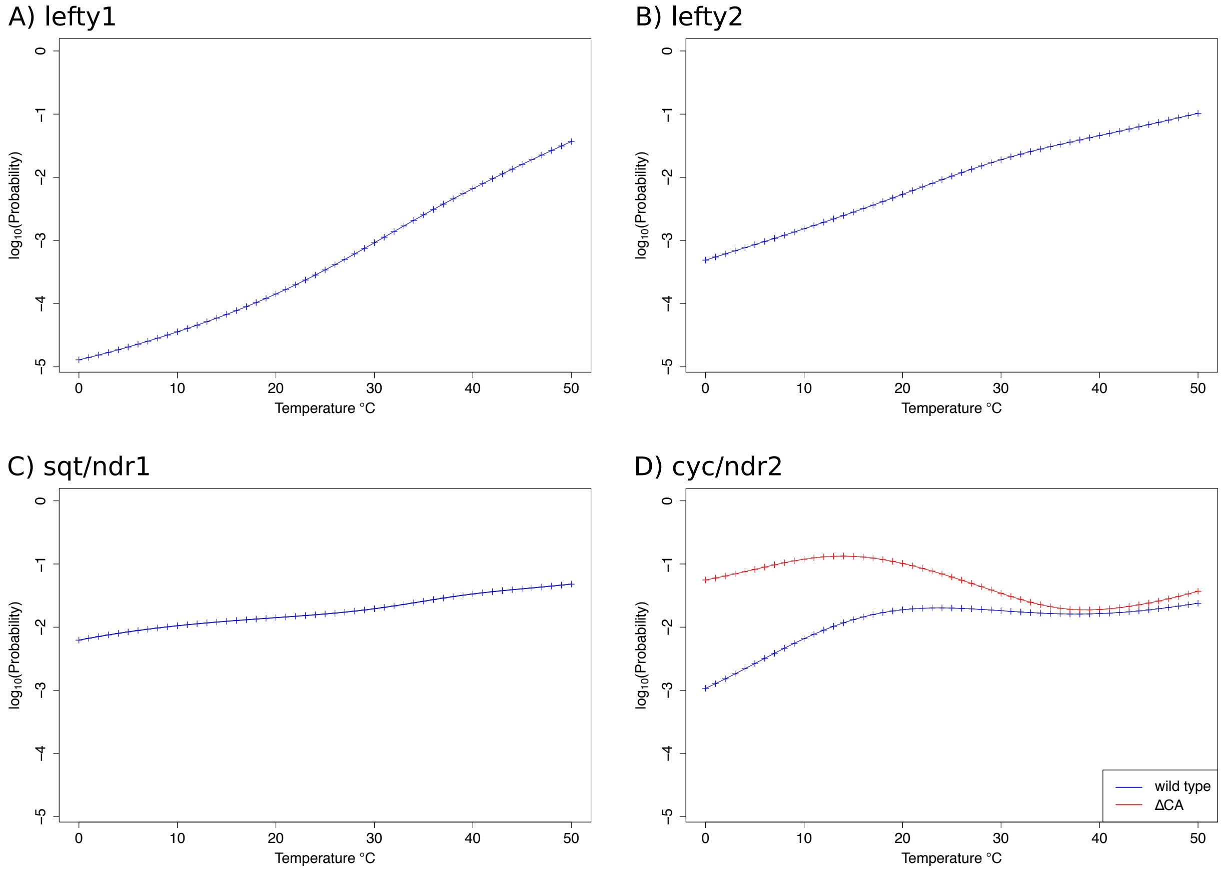

Figure S5: Probability for DLE stem loop formation in dependence of temperature.

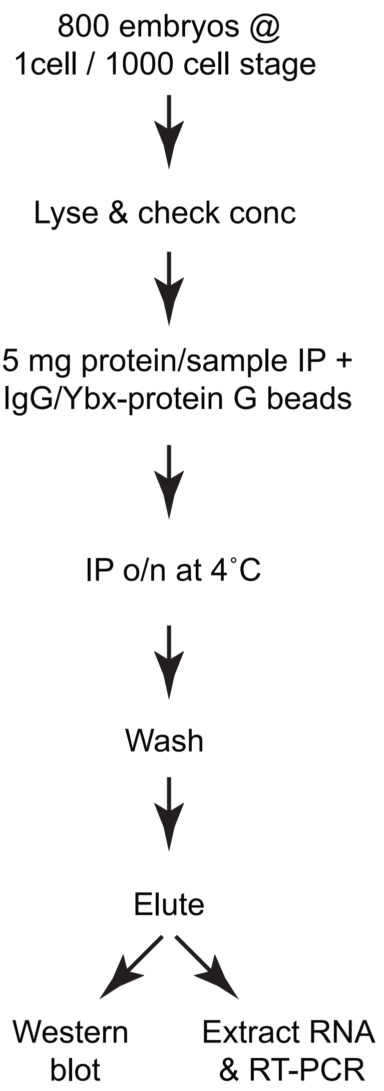

Figure S6: Schematic of RNA immunoprecipitations using zebrafish embryo lystes from 1-cell or 1000-cell embryos.

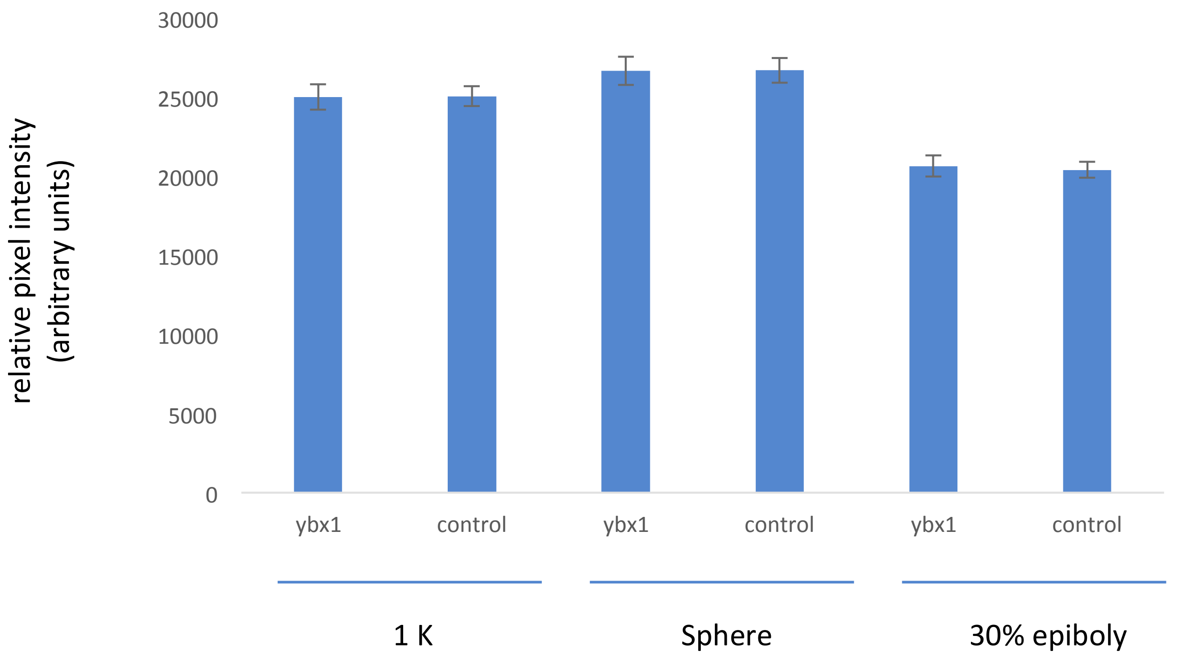

Figure S7: The injected internal standard (Rhodamine dextran) used for quantification of Lefty1-GFP expression shows uniform expression in stage matched control and *ybx1* mutants at 1000 cell, sphere and 30% epiboly. N = 34, error bars = standard error of the mean (sem).

Supplementary references.

1. Lorenz, R., Bernhart, S.H., Honer Zu Siederdissen, C., Tafer, H., Flamm, C., Stadler, P.F. and Hofacker, I.L. (2011) ViennaRNA Package 2.0. *Algorithms Mol Biol*, **6**, 26.
2. Andronescu, M., Condon, A., Hoos, H.H., Mathews, D.H. and Murphy, K.P. (2007) Efficient parameter estimation for RNA secondary structure prediction. *Bioinformatics*, **23**, i19-28.
3. Hecker, N., Christensen-Dalsgaard, M., Seemann, S.E., Havgaard, J.H., Stadler, P.F., Hofacker, I.L., Nielsen, H. and Gorodkin, J. (2015) Optimizing RNA structures by sequence extensions using RNAcop. *Nucleic Acids Res*, **43**, 8135-8145.
